# Prophylactic TLR9 stimulation reduces brain metastasis through microglia activation

**DOI:** 10.1371/journal.pbio.2006859

**Published:** 2019-03-28

**Authors:** Amit Benbenishty, Meital Gadrich, Azzurra Cottarelli, Alisa Lubart, David Kain, Malak Amer, Lee Shaashua, Ariella Glasner, Neta Erez, Dritan Agalliu, Lior Mayo, Shamgar Ben-Eliyahu, Pablo Blinder

**Affiliations:** 1 School of Psychological Sciences, Tel Aviv University, Tel Aviv, Israel; 2 Neurobiology Department, Tel Aviv University, Tel Aviv, Israel; 3 Sagol School of Neuroscience, Tel Aviv University, Tel Aviv, Israel; 4 School for Molecular Cell Biology & Biotechnology, Tel Aviv University, Tel Aviv, Israel; 5 Department of Neurology, Columbia University Medical Center, New York, New York, United States of America; 6 Department of Pathology, Sackler School of Medicine, Tel Aviv University, Tel Aviv, Israel; 7 The Lautenberg Centre for General and Tumor Immunology, The Hebrew University Hadassah Medical School, Jerusalem, Israel; National Cancer Institute, United States of America

## Abstract

Brain metastases are prevalent in various types of cancer and are often terminal, given the low efficacy of available therapies. Therefore, preventing them is of utmost clinical relevance, and prophylactic treatments are perhaps the most efficient strategy. Here, we show that systemic prophylactic administration of a toll-like receptor (TLR) 9 agonist, CpG-C, is effective against brain metastases. Acute and chronic systemic administration of CpG-C reduced tumor cell seeding and growth in the brain in three tumor models in mice, including metastasis of human and mouse lung cancer, and spontaneous melanoma-derived brain metastasis. Studying mechanisms underlying the therapeutic effects of CpG-C, we found that in the brain, unlike in the periphery, natural killer (NK) cells and monocytes are not involved in controlling metastasis. Next, we demonstrated that the systemically administered CpG-C is taken up by endothelial cells, astrocytes, and microglia, without affecting blood-brain barrier (BBB) integrity and tumor brain extravasation. In vitro assays pointed to microglia, but not astrocytes, as mediators of CpG- C effects through increased tumor killing and phagocytosis, mediated by direct microglia-tumor contact. In vivo, CpG-C–activated microglia displayed elevated mRNA expression levels of apoptosis-inducing and phagocytosis-related genes. Intravital imaging showed that CpG-C–activated microglia cells contact, kill, and phagocytize tumor cells in the early stages of tumor brain invasion more than nonactivated microglia. Blocking in vivo activation of microglia with minocycline, and depletion of microglia with a colony-stimulating factor 1 inhibitor, indicated that microglia mediate the antitumor effects of CpG-C. Overall, the results suggest prophylactic CpG-C treatment as a new intervention against brain metastasis, through an essential activation of microglia.

## Author summary

Brain metastases are prevalent and often terminal. Thus, reducing their occurrence could markedly improve cancer outcome. We show that systemic prophylactic and perioperative administration of a TLR9 agonist, CpG-C, reduced metastatic growth in experimental and spontaneous brain metastasis models, employing mouse and human tumors. CpG-C was taken up in the brain without affecting blood-brain barrier integrity and tumor extravasation. In vitro assays, imaging flow cytometry, and intravital imaging pointed to microglia as mediators of CpG-C effects through contact-dependent tumor killing and phagocytosis, corresponding with in vivo mRNA profile. In vivo depletion studies proved that microglia, but not NK cells or monocytes, mediated the beneficial effects of CpG-C, also hindered by blocking microglial activation. In toto, perioperative treatment with CpG-C should be considered clinically relevant.

## Introduction

Ten to twenty percent of cancer patients develop brain metastases, commonly as the final stage of cancer progression, with lung and melanoma cancers having the highest incidence (40%–50% and 30%–50%, respectively) [1]. Therapies include surgery and radiation; however, both treatments result in only a modest survival advantage and are associated with cognitive impairments [2]. Chemotherapy is often inefficient due to impermeability of the blood-brain barrier (BBB) [1], and as it often induces astrocyte-derived tumor-protecting responses [3]. Overall, the efficacy of currently available treatments for brain metastasis is extremely limited, making it a deadly disease with a short survival period [2]. Thus, prophylactic approaches against the establishment of brain metastasis, or early elimination of brain micrometastases, could prove key in treating cancer [2,4,5], and even more so given ongoing progression in early cancer detection and prevention of peripheral metastases.

In recent years, immune modulation using toll-like receptor (TLR) agonists has been given much attention as a therapeutic approach against primary tumors and metastasis [6]. Specifically, the TLR9 agonists CpG-oligodeoxynucleotides (ODNs) are being explored in a wide range of tumor types, both as single agents and as adjuvants [7,8], and are being tested in several clinical trials [9]. In various animal models, CpG-ODN treatment was shown to reduce mammary lung metastases by eliciting antitumor natural killer (NK) activity [9], and even results in rapid debulking of large tumors by macrophage stimulation [10]. Employed prophylactically, CpG-ODNs were shown to markedly improve resistance to experimental and spontaneous peripheral metastasis of mammary [11], colon [12], and melanoma [13] tumors.

Given the low success rate of treatments against established brain metastases [1], prophylactic treatment against metastatic brain disease may be key to improve survival rates [4]. Such treatment should be given chronically between primary tumor diagnosis and until several days/weeks following tumor removal. This time frame includes the short perioperative period, which was shown to constitute a high-risk period for initiation or accelerated progression of metastasis [14]. Prophylactic treatment should be especially advantageous in patients with primary tumors that have high potential of developing brain metastases, such as lung, melanoma, and breast cancers [15]. In fact, the concept of prophylactic treatment against brain metastasis is not unprecedented and is routinely practiced in the clinic. Small-cell lung cancer (SCLC) patients without detectable brain metastases often undergo prophylactic whole brain radiation therapy, thereby reducing occurrence of brain metastases and improving survival [16,17]. However, to implement a prophylactic approach against brain metastases in a wider range of patients, a less toxic [18] treatment is required. TLR9 stimulation using CpG-ODNs is particularly well suited to meet this need, as it has negligible toxicity in humans [19–21], and has already promising preclinical outcomes in other organs [10–13]; therefore, it should also be considered a potential prophylactic approach against the establishment of brain metastases.

In the brain, TLR9 is expressed on neurons, astrocytes, microglia, and endothelial cells [22,23]. Recent studies suggest that TLR9 signaling plays a key role in cerebral ischemia [24], cerebral malaria [25], Alzheimer’s [26,27], and seizures [28], pointing to its key role in healthy brain function and neuro-immune modulation. Notably, intracerebral [29,30] and retro-orbital [31] administrations of CpG-ODNs were shown to hinder growth of glioma [31] and intracranially injected melanoma cells [29,30]. Importantly, CpG-ODN yielded promising initial outcomes with minimal toxicity in a few Phase I/II clinical trials of recurrent [20,21] and de novo [19] glioblastoma, when injected into tumor-excised lesions. However, as a prophylactic measure against potential brain metastasis, CpG-ODNs would need to be administered systemically, provided they can cross the BBB.

Here, we assessed the efficacy of a systemic administration of CpG-C as a prophylactic treatment for brain metastasis using three preclinical mouse models, including experimental metastasis of syngeneic and of human lung carcinoma, and spontaneous metastasis of syngeneic melanoma. Importantly, the inoculation methodologies and imaging approaches implemented here preserve intact both the neuro-immune niche and brain hemodynamics intact: crucial factors affecting metastatic early stages [32]. We demonstrate that acute and chronic prophylactic treatments result in reduced brain metastasis in both sexes and across ages. Notably, we found that NK cells and monocytes/macrophages do not take part in the initial steps of the metastatic process in the brain, nor do they mediate the effects of CpG-C, as opposed to their role in peripheral organs. We establish that peripherally administered CpG-C crosses into the brain parenchyma without affecting BBB permeability, and that cerebral endothelial cells, astrocytes, and microglia take it up. We found that CpG-C–activated primary microglial cells and the N9 microglial cell line (but not primary astrocytes) eradicate tumor cells in vitro through direct contact, by increasing microglia cytotoxicity and phagocytic potential. Importantly, we demonstrate in vivo that following systemic CpG-C treatment microglia cells contact, kill, and phagocytize tumor cells during the early stages of invasion into the brain. Blocking microglia activation or depleting them abolished the beneficial effects of CpG-C. Taken together, our results point to CpG-C as an important potential prophylactic treatment against brain metastasis through direct activation of microglia.

## Methods

### Cell preparation and in vitro experiments

#### Tumor cells

Mouse D122 Lewis lung carcinoma (LLC) and mouse Ret melanoma cells [33] (both syngeneic to C57BL/6J mice), and human PC14-PE6 adenocarcinoma cells were cultured in complete media (RPMI 1640 supplemented with 10% fetal bovine serum and 1% penicillin/streptomycin; Biological Industries). D122-LLC and PC14-PE6 cells (kindly provided by Prof. Isaiah Fidler) were double labeled with mCherry and Luc2 (pLNT/Sffv-MCS/ccdB plasmid was kindly provided by Prof. Vaskar Saha), and Ret melanoma cells were labeled with mCherry. For two-photon experiments, D122 cells were infected with pLVX-tdTomato-N1 (Clontech). For experiments assessing cancer cell retention, cultured tumor cells were incubated with ^125^IUDR (iododeoxyuridine) during the last 24 hours of proliferation. Before injection, cells were washed and harvested (0.25% trypsin-EDTA; Invitrogen) at approximately 90% confluence, resuspended in PBS supplemented with 0.1% BSA (Biological Industries), and kept on ice throughout the injection procedures, completed within 3 hours of cell harvesting. More than 95% of cells were vital throughout the injection period.

#### Primary cultures (Fig 5A–5D)

We followed the mild trypsinization procedure [34]. Briefly, cortices of 1–3-day-old C57BL/6J pups were cultured in 12-well plates at a concentration of 4 × 10^5^ cells/well. After 18–25 days, astrocytes were removed with trypsin and cultured on separate plates. Cultures were used within 4 days from trypsinization.

#### Microglial N9 cell line [35] (Fig 5E–5I)

Cells were cultured in complete media (see above) at a concentration of 4 × 10^4^ cells/well.

### Experimental procedures

Cultures were subjected to 100 nM/L of CpG-C or non-CpG ODN (control) for 24 hours, and media was harvested for conditioned-media experiments (Fig 5B, 5D and 5G). Cultures were washed twice and supplied with fresh media. For contact cocultures experiments (Fig 5A, 5C, 5E and 5H), D122 cells were plated on top of the microglia cultures for six hours, following which cell-lysis (primary cultures; Fig 5A and 5C), bioluminescence imaging (N9 cultures; Fig 5E), or FACS analysis (Fig 5H) assays were conducted. For no-contact cocultures (Fig 5I), D122 cells were plated on 13-mm coverslips and placed on top of 2-mm-thick custom-made polydimethylsiloxane 11-mm rings over the microglial cultures (sharing the same media for six hours). For conditioned-media experiments (Fig 5F), D122 cultures were washed and supplied with fresh or conditioned media harvested from microglia or astrocyte cultures, for six hours.

### Cell-lysis assay (Fig 5A–5D)

Standard cytotoxicity assay was conducted as previously described [36]. Briefly, we used two concentrations of ^125^IUDR-labeled D122 cells in 12-well plates (1 × 10^4^ and 2 × 10^4^ cells/well). The radioactive signal from the media was measured using a gamma counter (2470, PearkinElmer). Specific killing was calculated as follows:

$$\left[\frac{sample\;release-spontaneous\;release}{maximal\;release-spontaneous\;release}\right]\times 100$$

### In vitro bioluminescence viability assay (Fig 5E–5G)

N9 cells were plated in 24-well plates (40 × 10^3^ cells/well) and treated with CpG-C or non-CpG ODN for 24 hours. We used two concentrations of Luc2-labeled D122 cells in 24-well plates (1.6 × 10^4^ and 3.2 × 10^4^ cells/well). D-luciferin (30 mg/mL, 10 μL) was mixed in each well and the bioluminescence signal was immediately measured for two minutes using Photon Imager and analyzed with M3 Vision (Biospace Lab).

Lysis and bioluminescence assessments were repeated in at least three separate experiments, each one conducted in quadruplicates or more.

### Apoptosis quantification (Fig 5H and S9 Fig)

Cocultures of N9 and D122 cells were stained for annexin V (88-8005-72, eBioscience), as per manufacturer’s instructions. We quantified the percent of annexin V–positive (apoptotic) cells from all mCherry-positive (D122) cells using FACScan (Becton Dickinson).

### Phagocytosis assay (Fig 5I)

N9 cells were plated in 96-well plates (30 × 10^3^ cells/well) and treated with CpG-C or non-CpG ODN for 24 hours. Cultures were washed twice and plated with pHrodo Red Zymosan Bioparticles (Thermo Fisher Scientific) conjugate for phagocytosis, according to the manufacturer’s instructions. These particles become fluorescent only after phagocytized into the lysosomes. Fluorescence was measured with Synergy HT (BioTek) microplate reader at 545/585 (Ex/Em) every 30–60 minutes thereafter (up to six hours). The maximum difference between experimental groups was used for statistical analysis.

### Scratch assay (S8 Fig)

N9 cells were plated in 96-well plates (30 × 10^3^ cells/well) and treated with CpG-C or non-CpG ODN as above. Plates were washed and fresh media was added. Confluent cultures were scratched (700 μm) using the IncuCyte Zoom system (Essen BioScience), washed, and imaged once every 2 hours for 28 hours.

### Ethics statement

All studies were approved by the Tel Aviv University (protocols numbers 04-16-039, 10-19-002, 10-13-002, and 10-13-015) and Columbia University (protocol number AAAX3452) corresponding ethics committees for animal use and welfare and were in full compliance with IACUC guidelines.

Male and female mice were used. Animals were housed under standard vivarium conditions (22 ± 1°C, 12-hour light/dark cycle, with ad libitum food and water). Anesthesia was first induced by 5% isoflurane and then maintained on 1.5%–2% throughout the procedures. When anesthetized, core body temperature of animals was maintained at 37°C. Animals placed with a cranial window received a single injection of carprofen (5 mg/kg; intraperitoneal [i.p.]) following the surgical procedure. Animals losing 10% of body weight were euthanized. For euthanasia of animals, we used excess CO_2_ or sodium pentobarbital (200 mg/kg; i.p.).

### Internal carotid artery inoculation of tumor cells

**Fig 1:**
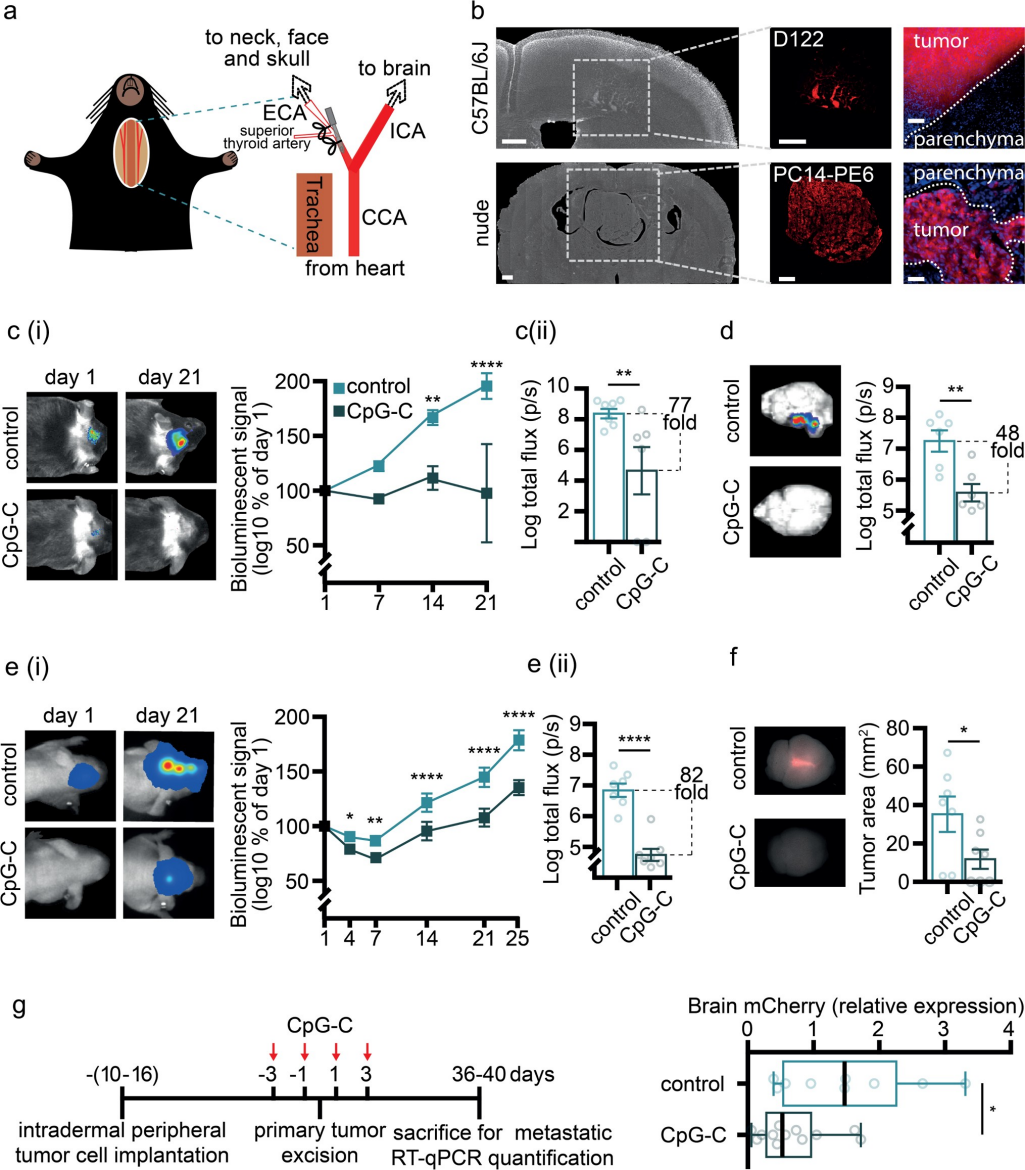
A single systemic prophylactic treatment with CpG-C results in long-term reduction of metastatic burden in the brain. (**a**) In the experimental metastasis models, we used the aECAi approach [32] for injection of tumor cells (see Methods): a method that improves brain targeting and preserves cerebral hemodynamics. (**b**) Histological images of D122 brain metastases from C57BL/6J mice on day 21 post–tumor inoculation show well-demarcated metastases, as well as vessel co-option growth. PC14-PE6 brain metastases from nude animals on day 25 post–tumor inoculation show large, well-demarcated metastases. Scale bar is 500 μm for the images on the left and middle, and 50 μm for the images on the right. (**c-f**) A single prophylactic systemic (i.p.) injection of 4 mg/kg CpG-C resulted in reduced growth of experimental brain metastases, as indicated by bioluminescence and fluorescence imaging. (**c**) C57BL/J6 mice injected with syngeneic D122 tumor cells and pretreated with CpG-C had reduced tumor burden compared with control animals, becoming significant on day 14 (**ci**; *n* = 6–7; F_(1,11)_ = 19.02, *p* = 0.0011) and reaching a 77-fold difference in total flux on day 21 (two-tailed Mann–Whitney *U* = 3, *p* = 0.0082; **cii**). Interestingly, in two CpG-C–treated animals, the bioluminescent signal gradually decreased and disappeared on day 21. (**d**) Ex vivo bioluminescence imaging of the brains from the syngeneic model indicated a 48-fold reduced tumor burden (total flux) in CpG-C–treated animals (*n* = 6; two-tailed unpaired Student *t* test, *t*_(10)_ = 3.722, *p* = 0.0040). (**e**) Athymic nude mice injected with human (xenograft) PC14-PE6 tumor cells and pretreated with CpG-C had reduced tumor burden compared with control animals, becoming significant on day 4 (**e.i**; *n* = 7; F_(1,12)_ = 77.45, *p* < 0.0001) and reaching a 82-fold difference in total flux on day 25 (two-tailed unpaired Student *t* test, *t*_(12)_ = 7.090, *p* < 0.0001; **e.ii**). (**f**) Using Maestro fluorescence imaging, a reduction in brain tumor burden (i.e., tumor area) was evident in the human xenograft model in CpG-C–treated animals (*n* = 7; two-tailed Mann–Whitney *U* = 8, *p* = 0.0373). (**g**) (**left**) Timeline for spontaneous melanoma brain metastasis model [33] (see Methods). (**right**) CpG-C treatment during seven perioperative days resulted in reduced micrometastases in the brain (measured by mCherry mRNA expression; *n* = 9 and *n* = 12 for control and CpG-C, respectively; two-tailed unpaired Student *t* test, *t*_(19)_ = 2.278, *p* = 0.0345). The background of images was manually removed. Box plot whiskers represent minimum–maximum range. See S1 Fig for comparison of bioluminescent signal at day 1. The underlying data for this figure can be found in S1 Data. aECAi, assisted external carotid artery inoculation; CCA, common carotid artery; ECA, external carotid artery; ICA, internal carotid artery; i.p., intraperitoneal; RT-qPCR, real-time quantitative polymerase chain reaction.

Tumor cells were injected using the assisted external carotid artery inoculation (aECAi; Fig 1A), as previously described [32]. Briefly, mice were anesthetized and the external carotid artery (ECA) uncovered. A 6–0 silk-suture ligature was loosely placed around the ECA proximal to the bifurcation from the common carotid artery (between the superior thyroid artery and the bifurcation). A second ligature was tied on the ECA distal to the bifurcation. A NANOFIL-100 (WPI) syringe with a 34G beveled needle was mounted to a micromanipulator (M33, Sutter). The needle was inserted slowly into the lumen of the ECA and advanced to the point of bifurcation. The first ligature was tied around the needle, and 1 × 10^5^ cells in PBS (100 μL) were slowly infused into the internal carotid artery. The needle was then removed, the ligature quickly tied, and the skin sutured. Total time for the complete procedure is 10 minutes.

### Spontaneous melanoma brain metastasis

For a spontaneous brain metastasis model (Fig 1G), we used the Ret-melanoma model we have recently established and validated [33]. Briefly, mice were anesthetized by isoflurane, and a total of 5 × 10^5^ (50 μL) Ret-mCherry–sorted (RMS) cells in a 1:1 suspension of PBS with growth factor–reduced Matrigel (356231, BD Biosciences) were injected subdermally to the right dorsal side, rostral to the flank, with a 29G insulin syringe (BD Biosciences). Tumors were measured four times weekly by calipers. Tumor volumes were calculated using the formula X^2^ × Y × 0.5 (X = smaller diameter, Y = larger diameter). We aimed to remove the tumor at a size of approximately 500 mm^3^. Therefore, and based on our experience, once tumors reached a size of about 125 mm^3^, mice were given two injections of CpG-C or PBS (control group) every other day. One day later (i.e., three days following the first CpG-C treatment and one day following the second CpG-C treatment), tumor sizes were verified (meeting our expectations, with no differences between treatment groups) and immediately removed. The last tumor removal was carried out six days after the first removal. For tumor excision, mice were anesthetized with isoflurane, and an incision, medial to the tumor, was made in the skin. Tumors were detached from inner skin with clean margins to prevent recurrence. Tumor-associated connective tissue and blood vessels were detached, and the incision was sutured. Primary tumors were sectioned and measured with no difference identified at excision time (S2 Fig). Mice were weighed weekly and monitored for relapse. Nine weeks after last tumor excision, mice were deeply anesthetized with isoflurane and transcardially perfused with cold PBS. Brains and lungs were harvested, macroscopically examined for abnormal lesions, and flash-frozen in liquid nitrogen. RNA was isolated using EZ-RNA II kit (20-410-100, Biological Industries) according to the manufacturer’s instructions. Whole organs were homogenized in denaturation solution A in M tubes (130-096-335, Milteny Biotec) by gentleMACS dissociator (Milteny Biotec). Reverse transcription was performed with qScript (95047–100, Quanta Biosciences). RT-qPCR analyses were conducted using PerfeCTa SYBR Green FastMix, ROX (95073-012-4, Quanta Biosciences) with primers for Hprt (F sequence: GCGATGATGAACCAGGTTATGA; R sequence: ATCTCGAGCAAGTCTTTCAGTCCT) and mCherry (F sequence: GAACGGCCACGAGTTCGAGA; R sequence: CTTGGAGCCGTACATGAACTGAGG).

In all analyses, expression results were normalized to *Hprt*. RQ (2^−ΔΔCt^) was calculated.

Of the 50 animals initially injected with tumor cells, two animals did not develop primary tumors and were withdrawn from the experiment; of the remaining 48 animals, 28 animals were treated with CpG-C and 20 with PBS (control). Twenty-two animals (45% of control and 46% of CpG-C treated) died during the period between tumor excision and the day of tissue collection, leaving 15 CpG-C–treated animals and 11 control animals. In three CpG-C animals and two control animals, we did not detect mCherry RNA in the brains. The herein development of primary tumor and metastases is expected based on our previous studies in this tumor model [33]. Tumor burden was compared in animals bearing brain micrometastasis.

### ODN treatment

CpG-C, CpG-C-FITC, and CpG-C-TAMRA (ODN 2395: 5′-TCGTCGTTTTCGGCGCGCGCCG-3′) with a phosphorothioate backbone and non-CpG ODN (ODN 2137: 5′-TGCTGCTTTTGTGCTTTTGTGCTT -3′), endotoxin-free, were purchased from Sigma-Aldrich. Two different controls were used: PBS and non-CpG ODN, which lacks C-G motifs (counterbalanced within experiments with no differences in results). Both CpG-C variants and non-CpG ODN were diluted in PBS and administered intraperitoneally (100 μL) at a dose of 0.4 or 1.2 mg/kg (S3 Fig), or 4mg/kg (all in vivo experiments). No differences were found between PBS- and non-CpG ODN–treated animals, and therefore they were combined in the statistical analyses (S7 Fig).

### Depletion of NK cells and monocytes/macrophages

**Fig 2:**
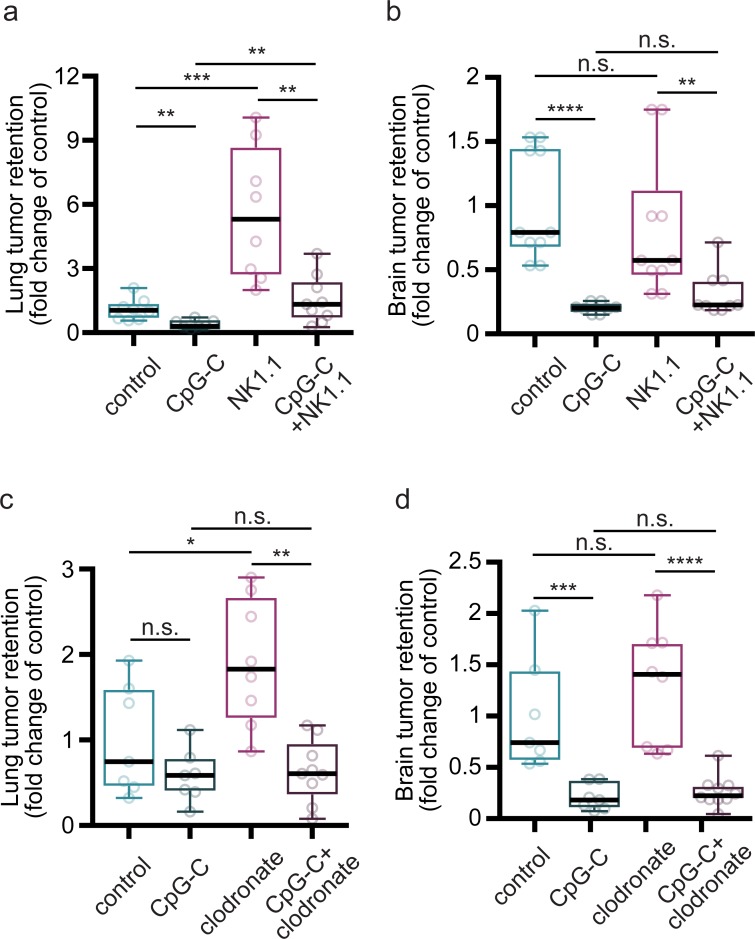
The effects of CpG-C on brain metastases are not mediated by NK cells or monocytes. (**a**) Depletion of NK cells using NK1.1 antibody resulted in a 5-fold elevation in D122 LLC tumor retention in the lungs (*n* = 8; *t*_(14)_ = 4.4781, *p* = 0.0001), and partially blocked the beneficial effects of CpG-C (*n* = 8; *t*_(14)_ = 1.1517, *p* = 0.0038), evident in naïve animals (*n* = 8; *t*_(14)_ = 0.7002, *p* = 0.0019). (**b**) In brains of the same animals, NK depletion had no effect on tumor retention (*n* = 8; *t*_(14)_ = 0.1894, *p* = 0.3935), nor mediated the beneficial effects of CpG-C (*n* = 8; *t*_(14)_ = 0.1099, *p* = 0.0811), evident in both naïve (*n* = 8; *t*_(14)_ = 0.7973, *p* < 0.0001) and NK-depleted animals (*n* = 8; *t*_(14)_ = 0.4979, *p* = 0.0056). (**c**) Depletion of monocytes using clodronate liposomes resulted in increased lung tumor retention of D122 LLC cells (*n* = 7–8; *t*_(13)_ = 0.9072, *p* = 0.0292), an effect rescued by CpG-C (*n* = 8–9; *t*_(15)_ = 1.2700, *p* = 0.0003), indicating that lung tumor retention is mediated by monocytes, while they do not mediate the effects of CpG-C. (**d**) In brains of the same animals, monocyte depletion did not affect tumor retention (*n* = 7–8; *t*_(13)_ = 0.3028, *p* = 0.3081), and CpG-C reduced tumor retention in naïve (*n* = 7; *t*_(12)_ = 0.7910, *p* = 0.0006) and monocyte-depleted animals (*n* = 8–10; *t*_(16)_ = 1.0377, *p* = 0.0001). Two-way permutations were used for the above analyses. Box plot whiskers represent minimum–maximum range. The underlying data for this figure can be found in S1 Data. LLC, Lewis lung carcinoma; NK, natural killer; n.s., nonsignificant.

For depletion of NK cells (Fig 2A and 2B), anti-NK1.1 monoclonal antibodies (mAbs) were intraperitoneally administered (4 mg/kg) 24 hours before tumor cell injection. mAb (12E7) against human cluster of differentiation (CD) 99 served as control. Antibodies [38] were kindly provided by Prof. Ofer Mandelboim (The Hebrew University of Jerusalem, Israel). To verify depletion of NK cells, blood was collected from animals during tissue collection, and prepared for staining with NK1.1 FITC (eBioscience) and NKp46 PE (BioGems) [39]. FACS analysis indicated >90% depletion (S4 Fig).

For monocytes/macrophages depletion (Fig 2C and 2D), we administered clodronate liposomes (ClodronateLiposomes.org) intravenously (200 μL) 24 hours before tumor cell injection. PBS liposomes served as controls. To verify depletion of monocytes, but not microglia, blood was sampled, animals were perfused, and brains were collected. Samples were prepared for staining with F4/80 FITC and CD11b APC (BioGems). FACS analysis indicated >85% depletion of monocytes/macrophages, without affecting microglia viability (S4 Fig).

### Microglia inactivation (Fig 7A and 7B)

To block microglia activation and transition into an inflammatory state [40], minocycline hydrochloride (Sigma-Aldrich) was administered intraperitoneally at a dose of 40 mg/kg (200 μL) at 48, 32, and 24 hours before tumor cell injection.

### Depletion of microglia cells (Fig 7C)

For depletion of microglia cells, mice were administered the dietary inhibitor of colony stimulating factor-1 receptor (CSF1R), PLX5622 (1,200 mg/kg chow; provided by Plexxikon and formulated in AIN-76A standard chow by Research Diets) for 18 days, resulting in near-complete elimination of microglia cells [41]. AIN-76A standard chow served as control (Research Diets).

### Histology

C57BL/6J and athymic nude mice from the bioluminescence experiments were perfused with PBS supplemented with 30 U heparin (Sigma) and 4% paraformaldehyde (PFA) (EMS). Brains were harvested, fixed overnight in 4% PFA, and placed in 30% sucrose overnight. Thirty-micrometer sections (Leica SM 2000 microtome) were counterstained with DAPI (MP Biomedicals). Images of the sections were obtained using a fluorescent microscope (Olympus ix81; Fig 1B).

To visualize CpG-C uptake in the parenchyma, TAMRA-labeled CpG-C was injected to CX3CR1^GFP/+^ mice. Twenty-four hours later, animals were perfused and brains fixed and sectioned. Astrocytes were stained using a primary anti-GFAP antibody (1:800; Invitrogen) and endothelial cells with anti-CD31 (PCAM-1) antibody (1:500; Santa Cruz). A secondary Alexa 647 antibody (1:600; Invitrogen) was used, coupled with DAPI (1:1,000; ENCO) staining. Images of the sections were obtained using a Leica SP8 confocal microscope at 0.5-μm intervals using a ×63 (NA– 1.4) oil immersion objective.

### Imagestream

Preparation of tissue for ImageStream (MK II; Amnis) FACS analysis—mice were perfused with PBS supplemented with 30 U heparin (Sigma-Aldrich) and brains removed. Brains were mechanically minced, suspended in a solution containing collagenase (0.1% w/v; Worthington) and dispase (0.2% w/v; Roche) for 20 minutes and then in DNAse (Sigma-Aldrich) for 20 minutes, and suspensions were passed through a 70-μm filter. Fatty tissue was removed using Percoll (Sigma-Aldrich), and cells were resuspended in PBS supplemented with 1% EDTA (Sigma-Aldrich), 0.01% NaN_3_ (Sigma-Aldrich), and 1% FBS (Biological Industries). In each experiment, 1 × 10^4^ events were collected and analyzed using Amnis IDEAS software (Version 6.2). Analysis gates were manually corrected based on images of the events. Internalization was quantified automatically using the software’s internalization wizard (Figs 6F–6H and 7B; S9 Fig).

**Fig 3:**
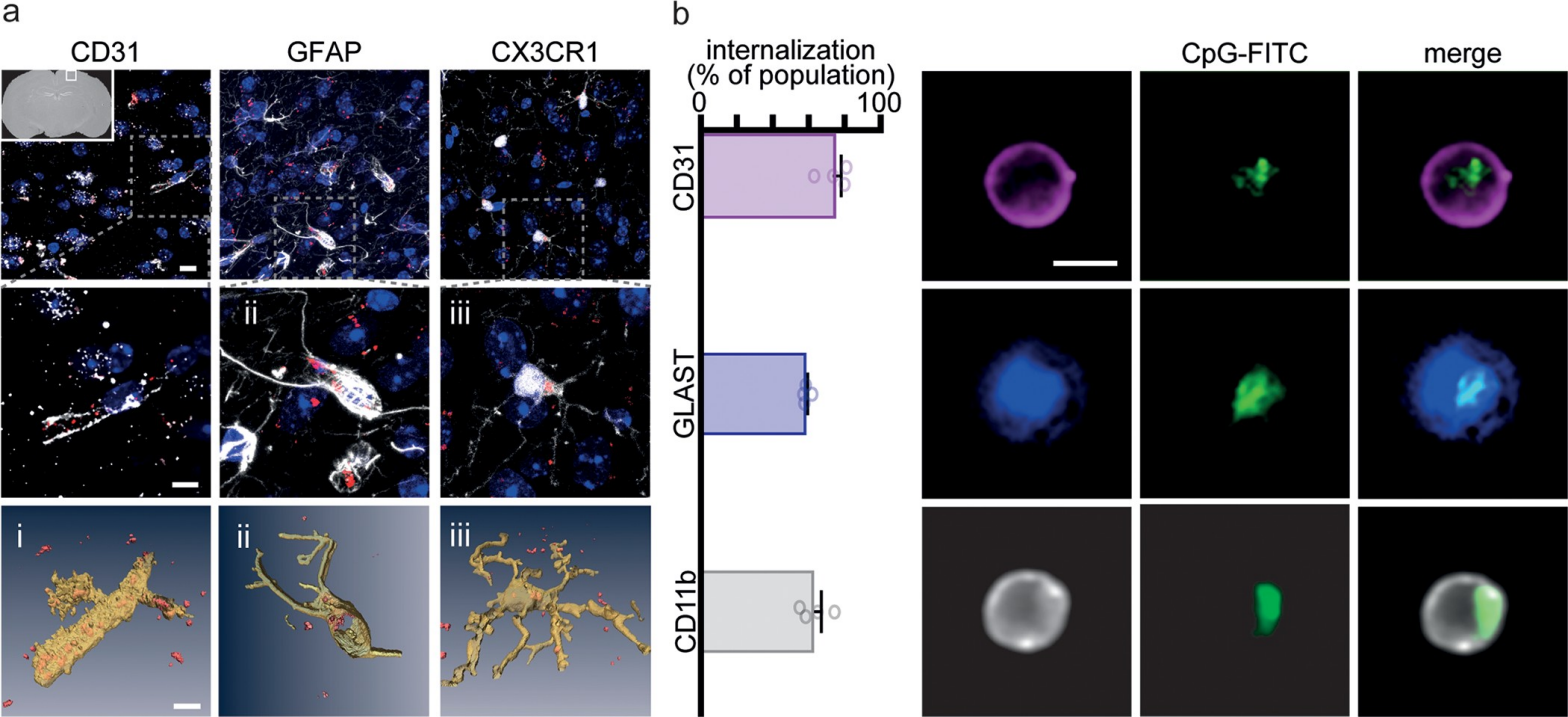
CpG-C infiltrates the brain and is internalized by endothelial cells, astrocytes, and microglia. (**a-b**) TAMRA-labeled CpG-C was injected intraperitoneally; 24 hours later, brains were perfused, and CpG-C internalization in endothelial cells (CD31), astrocytes (GFAP and GLAST), and microglia (CX3CR1 and CD11b) was visualized in histological sections using confocal microscopy (**a**; top panels are 15-μm z-max projections in the primary somatosensory cortex, and lower panels are partial reconstructions) and quantified using ImageStream FACS analysis (**b**). The majority of each of the three cell populations internalized CpG-C, indicating that CpG-C crosses the BBB into the parenchyma (*n* = 4). Scale bar is 5 μm. Data presented as mean (±SEM). The underlying data for this figure can be found in S1 Data, and our gating strategies are provided in S9 Fig. BBB, blood-brain barrier; CD, cluster of differentiation; CX3CR1, CX3C chemokine receptor 1; FACS, fluorescence-activated cell sorting; FITC, fluorescein isothiocyanate; GFAP, glial fibrillary acidic protein; GLAST, glutamate aspartate transporter; TAMRA, tetramethylrhodamine.

For quantification of CpG-C infiltration into the brain and its internalization by endothelial cells, astrocytes, and microglia (Fig 3), mice were injected with FITC-labeled CpG-C 24 hours before perfusion, and single cell suspensions were stained using anti-CD31 (PCAM-1) PE-Cy7 (eBioscience), Anti-GLAST (ACSA-1)-PE (MACS), and anti-CD11b APC (BioGems). To avoid GLAST staining of Bergmann glia, cerebellums were removed before preparation of the samples in this experiment. In each population, we quantified the percent of cells with internalized FITC.

### Lysotracker staining

Cultures of N9 cells grown on coverslips were treated with CpG-C-TAMRA for 24 hours and washed three times. LysoTracker Blue DND-22 (50 nM, Thermo Fisher Scientific) was applied for 30 minutes at 37°C, and coverslips were washed and mounted on slides. For staining of CpG-C uptake in vivo, a single cell suspension was prepared from CX3CR1^GFP/+^ mice treated with CpG-C-TAMRA as described in the ImageStream FACS analysis protocol herein. LysoTracker Blue DND-22 (50 nM, Thermo Fisher Scientific) was then mixed into the cell suspension for 30 minutes at 37°C, and cells were mounted on a cover glass and imaged with a Leica SP8 confocal microscope using a ×63 (NA– 1.4) oil immersion objective (Fig 5B). Similarly, for the cells extracted from the brains of animals, we imaged only green fluorescent protein (GFP)–positive cells (i.e., microglia).

### Claudin-5 continuity and IgG and biocytin-tetramethylrhodamine leakage quantification

**Fig 4:**
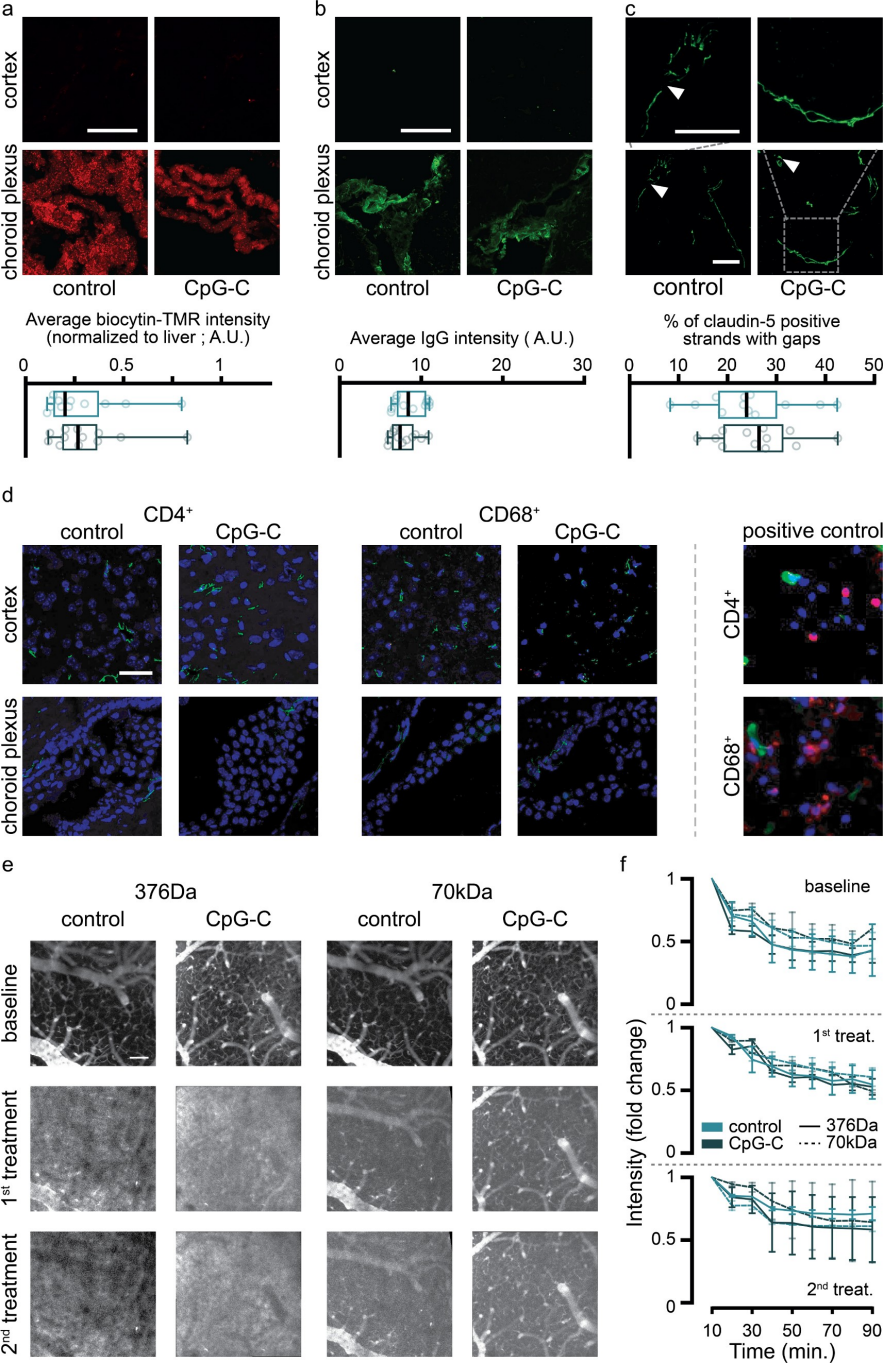
CpG-C does not affect BBB leakage or cellular permeability. (**a-c**) Biocytin-TMR was intravenously injected into animals expressing GFP under the claudin-5 promotor 24 hours following CpG-C or control treatment, and 90 minutes later brains were perfused and removed. Biocytin-TMR intensity (normalized to intensity levels in the liver, not shown) and IgG staining intensity were similar in CpG-C–treated and control animals (two-tailed unpaired Student *t* test, *t*_(22)_ = 0.3758, *p* = 0.7106; **a-b**). Moreover, no difference in number of gaps in claudin-5 strands was found between control and CpG-C–treated animals (two-tailed unpaired Student *t* test, *t*_(22)_ = 0.4283, *p* = 0.6726; **c**). (**d**) Brain sections of WT animals treated with CpG-C or PBS were stained for CD4 or CD68 24 hours following treatment. No infiltration of immune cells was detected (spinal cords of EAE mice served as positive controls; right panels). (**e-f**) Using two-photon imaging, we followed leakage of a low (NaF; 376 Da) and a high (Texas-Red; 70kDa) molecular weight dextrans. Intensities of representative images (**e**) were auto-adjusted in Fiji for display purposes only. No differences were found between control and CpG-C–treated animals at baseline (*p* = 0.8567 and *p* = 0.8421 for NaF and Texas-Red, respectively), following a single CpG-C treatment (*p* = 0.9243 and *p* = 0.2419 for NaF and Texas-Red, respectively), and following two CpG-C treatments (*p* = 0.4656 and *p* = 0.3918 for NaF and Texas-Red, respectively. See Methods for an explanation of the quantification; **f**). For (**a-c**), each sample consisted of an average of at least five images that were analyzed. Samples were taken from four different anatomical brain regions (cortex, midbrain, cerebellum, and hippocampus) in three mice/group (See S5 Fig for regional presentations). Scale bar is 50 μm. Box plot whiskers represent minimum–maximum range (**a-c**), and data in (**f**) are presented as mean (±SEM). The underlying data for this figure can be found in S1 Data. A.U., arbitrary units; BBB, blood-brain barrier; CD, cluster of differentiation; EAE, experimental autoimmune encephalomyelitis; GFP, green fluorescent protein; IgG, immunoglobulin G; TMR, tetramethylrhodamine; WT, wild type.

Tg eGFP-claudin-5 [37] mice were treated with CpG-C or PBS, and 23 hours later were injected with 1% biocytin-tetramethylrhodamine (TMR) (i.v., Life Technologies). One hour later, animals were perfused with PBS and 4% PFA. Brains and livers were harvested, fixed for six hours in 4% PFA at 4°C, and placed in 30% sucrose overnight at 4°C. Tissues were embedded in O.C.T (Sakura), sectioned (12 μm) using a Leica cryostat, and stained for eGFP (1:1,000; Life Technologies) and IgG (1:1,000; Invitrogen). Z-stacks of the sections were obtained with a Zeiss LSM700 confocal microscope using a water immersion ×40 objective (NA– 1.2), and maximum projections were created using Fiji (version 1.0). At least five images were used for quantification for each anatomical region. Biocytin-TMR and IgG intensity was quantified using Fiji software and normalized on fluorescence intensity in the liver (Fig 4A and 4B and S5 Fig). For quantification of gaps in tight junctions (Fig 4C and S5 Fig), we quantified the percentage of junctional strands showing at least one gap (defined as a discontinuity in eGFP-Caudin5 signal >0.4 μm) over the total number of junctional strands [37].

### Immune infiltration analysis

To test whether CpG-C affects immune cell infiltration into the brain, sections of PBS and CpG-C–treated mice were stained for CD68 (1:1,000; Abcam) and CD4 (1:200; Abcam). To assure we do not analyze immune cells arrested in the vessels, we costained slices with laminin (1:1,000; Sigma) to detect vessel walls. As a positive control we used spinal cord sections of experimental autoimmune encephalomyelitis (EAE) mice (refer to [42] for experimental procedure; Fig 4D).

### In vivo and ex vivo bioluminescent imaging (Fig 1C–1E)

To follow progression of tumor growth in vivo, we used an IVIS SpectrumCT (PerkinElmer) for the syngeneic model and Photon Imager (Biospace Lab) for the xenograft model. Briefly, mice were anesthetized and injected with D122-mCherry-Luc2 (C57BL/6J) or PC14-PE6-mCherry-Luc2 (athymic nude) cells. Imaging sessions were conducted on days 1, 4, 7, 14 and 21 following tumor cell administration (in the xenograft model, also on day 25). After the last in vivo imaging session in the syngeneic model, mice were killed, and brain and extracranial head tissue were rapidly imaged, separately. Notably, tissue from one control animal was lost in the final process. Each imaging session was preformed between 10 and 20 minutes following D-Luciferin sodium salt injection (30 mg/mL, 100 μL, i.p.; Regis Technologies), as this time frame exhibited maximal and steady intensity. Analysis was done using Living Image software (version 4.3.1) for the IVIS images data, and M3 vision for the Photon Imager data.

### Ex vivo fluorescence imaging (Fig 1F)

To quantify fluorescence in brains of athymic nude mice injected with PC14-PE6-mCherry-Luc2, animals were decapitated and brains were extracted immediately following the last imaging session. We used a Maestro spectral fluorescence imaging system (Cambridge Research and Instrumentation) and quantified the fluorescent signal using Maestro version 2.2 software. Regions of interest (ROIs) were drawn on each fluorescent signal to quantify the area of the fluorescent signal.

### Assessment of brain and peripheral organ retention of cancer cells

Mice were injected with 1 × 10^5 125^IUDR-labeled D122-LLC cells using the aECAi approach [32] and euthanized 24 hours later. Animals were transcardially perfused with 20 mL PBS supplemented with 30 U heparin (Sigma-Aldrich). Brain and lungs were collected, and radioactivity was measured using a gamma counter (2470, PearkinElmer; Figs 1F–1H, 2, 7A and 7C).

### Two-photon laser scanning microscopy

For two-photon microscopy measurements, CX3CR1^GFP/+^ and WT mice were implanted with a polished and reinforced thin-skull (PoRTS) window over the somatosensory cortex (window center at bregma minus 2 mm and 3 mm lateral to the midline), as previously described [43]. Importantly, this craniotomy does not elicit an inflammatory response [43]. Mice were then habituated to the imaging apparatus for 7 days to reduce procedural stress. CX3CR1^GFP/+^ animals were injected with 1 × 10^5^ tdTomato-labeled D122 cells. Before imaging, mice were injected with Alexa Fluor 633 hydrazide (2.5% w/v, i.v.; Invitrogen) for visualization of arteries [40]. Imaging sessions were initiated 2–4 hours after tumor cell inoculation, and at days 1, 2, 4 and 7, returning to the exact same location each session. Imaging was conducted at depths of 50–200 μm with a custom-modified two-photon laser-scanning microscope based on a Sutter MOM (Sutter) controlled through the ScanImage software (Vidrio Technologies). A Chameleon Ultra II (Coherent) provided the 80-MHz, 140-fs pulsed light used for imaging and laser photodamage.

For quantification of microglia-tumor cells interaction, 150-μm stacks were obtained and max projected every 10 μm. The number of contacts and internalization events in each stack were manually quantified blindly at 4 hours following tumor cell injection, and at days 1, 4, and 7 (Fig 6D and 6E). For imaging CpG-C uptake by microglia in vivo, baseline imaging of cortices of CX3CR1^GFP/+^ mice was performed at 890 nm, CpG-C-TAMRA was injected (4 mg/kg; 100 μL; i.p.), and 24 hours later mice were imaged again at the exact same locations (S6 Fig).

For longitudinal BBB assessment (Fig 4E and 4F), WT mice implanted with a PoRTS window were treated with four PBS or CpG-C injections every other day (similarly to the spontaneous melanoma brain metastasis experiment). BBB leakage dynamics of a low molecular weight dye (sodium fluorescein; NaF; 376 Da; Sigma-Aldrich) and of a higher molecular weight dye (Texas Red; 70 kDa; Invitrogen) was imaged simultaneously at 940 nm. The imaging session took place at baseline (before treatment), one day following the first CpG-C/control treatment, and one day following the last treatment. To this end, 10 minutes following dye injection, 100-μm stacks were taken every 10 minutes for a total of 90 minutes. For quantification of dye leakage, max-projections of each session were aligned using Fiji software (2.0) plugin *linear stack alignment with SIFT*. Eight vessels (four capillaries 5 μm and smaller, and four vessels 20–50 μm in diameter) were blindly selected manually, and average intensity was measured inside the vessel and adjacent to it (in the parenchyma). The ratio over time between the amount of dye inside and outside the vessels was computed (Fig 4F). For display purposes only, image contrast was automatically adjusted using the Fiji *autoadjust* display function, while measurements were taken directly from pixel values.

### Two-photon laser photodamage

In order to assess microglia reactivity, focal laser-induced thermal damage insults were performed as previously described [44] (S8 Fig). Briefly, CX3CR1^GFP/+^ mice underwent craniotomy, and three weeks later microglia, were imaged at 890 nm. A baseline stack (0–30-μm depth) was imaged and a small (15–20-μm) localized injury was achieved by focusing a two-photon laser beam (780 nm; 150 mW at the sample; about 1 μm in size) at 15-μm depth for two seconds. Stacks were imaged every two minutes for 40 minutes. Using Fiji software (1.0), maximum z-projections were turned into binary images. A 60-μm circle was drawn around the ablation area, and for each time point, the number of white pixels were counted inside the small circle (x(t)). For the baseline image, another 120-μm circle was drawn, and the white pixels in the ring between the two circles were counted (y(0)). Response was calculated as follows: *x*(*t*)−*x*(0)/*y*(0).

### Real-time quantitative polymerase chain reaction

In two independent experiments (Fig 8), male and female mature (4–6 months) CX3CR1^GFP/+^ mice were treated with CpG-C or non-CpG ODN/PBS. Twenty-four hours later, mice were perfused and brains were harvested and processed into a single cell suspension as described above. GFP-positive cells (microglia) were sorted (FACSAria IIU, BD Biosciences), and RNA was extracted with TRIzol (Invitrogen). cDNA was prepared and used for quantitative PCR, and the results were normalized to *Gapdh*. All primers and probes were purchased from Applied Biosystems, *Cd36* (Mm00432403_m1), *Cd47* (Mm00495006_m1), *Cd68* (Mm03047343_m1), Fas ligand (*Fasl*) (Mm00438864_m1), *Gapdh* (Mm99999915_g1), interferon gamma (*Inf-γ*) (Mm01168134_m1), interleukin (*Il*)*1-β* (Mm00434228_m1), *Il-6* (*Mm00446190_m1)*, Macrophage receptor with collagenous structure (*Marco*) (Mm00440250_m1), nitric oxide synthase 2 (*Nos2*) *(Mm00440502_m1)*, transmembrane protein 119 (*Tmem119*) (Mm00525305_m1), tumor necrosis factor (*Tnf*) (Mm00443258_m1), tumor necrosis factor superfamily member 10 (*Tnfsf10*) (Mm01283606_m1), and triggering receptor expressed on myeloid cells 2 (*Trem2*) (Mm04209424_g1). One control sample was removed as an outlier from statistical analysis of *Tnf* and *Inf-γ* (25 SEMs and 50 SEMs, respectively).

### Volumetric image display

Three-dimensional volumetric reconstruction of single cells (Fig 3A, S6 Fig) or fields of view (Fig 6C) were performed in a semi-automatic way using Amira software (Thermo Fisher Scientific). Auto-thresholding mode was initially used to detect the brightest object, which, depending on the experiment and the spectral channel under analysis, represented either cell soma or aggregates of labeled CpG-C. Cell morphology was partially reconstructed by manual labeling after thresholding.

### Statistical analysis

**Fig 5:**
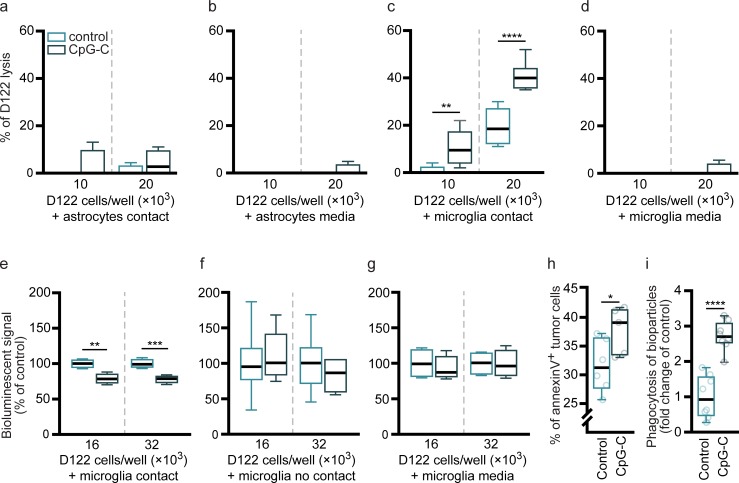
Microglia, but not astrocytes, mediate the effects of CpG-C in vitro. (**a-d**) Primary cultures of microglia and of astrocytes were subjected to 100 nM/L CpG-C or non-CpG ODN (control) for 24 hours. ^125^IUDR-labeled D122 cells were plated with the treated primary cultures or subjected to their conditioned media alone, and cytotoxicity (percent of D122 lysis) was assessed by measuring radioactivity in the media 8 hours later. Primary cultured astrocytes, subjected to CpG-C or non-CpG ODN, did not cause tumor cell lysis when in contact (F_(3,12)_ = 0.7755, *p* = 0.5298; **a**), nor did their conditioned media (F_(3,9)_ = 0.6923, *p* = 0.5794; **b**). In contrast, primary cultured microglia cells induced lysis in tumor cells, and treatment with CpG-C significantly increased it when in contact with tumor cells (F_(3,28)_ = 64.1, *p* < 0.0001; **c**), while their conditioned media had no effect (F_(3,9)_ = 0.6923, *p* = 0.5794; **d**). (**e-i**) The microglia cell line, N9, was subjected to CpG-C (see above). Luc2-mCherry–labeled D122 cells were plated with the N9 cultures with contact (**e**) or without contact (coculture; **f**), or with their conditioned media alone (**g**), and bioluminescent signal was measured, indicating viability of tumor cells. There was a reduced signal in tumor cells cultures that were in direct contact with N9 cells (F_(3,12)_ = 14.6, *p* = 0.0003; **e**), while no difference was evident in cocultures (no contact; F_(3,18)_ = 0.3535, *p* = 0.7872; **f**) or in cultures subjected to conditioned media (F_(3,12)_ = 0.1425, *p* = 0.9325; **g**). Two-tailed one-way ANOVA with Bonferroni multiple comparison correction was used for **a-g**. (**h**) Annexin V binding (a marker for early-stage apoptosis) was quantified in D122 cells cultured with pretreated N9 cells using FACS. Tumor cells cultured with N9 cells pretreated with CpG-C exhibited increased annexin V staining (compared with scrambled CpG-C; two-tailed Student *t* test for unpaired samples, *t*_(9)_ = 2.3060, *p* = 0.0465). (**i**) N9 cultures treated with CpG-C or non-CpG ODN (control) for 24 hours were washed and plated with pH-sensitive bio-particles to assess phagocytosis capacity. CpG-C–treated N9 cells exhibited a 3-fold increased phagocytic capacity (two-tailed Student *t* test for unpaired samples, *t*_(14)_ = 6.6960, *p* < 0.0001). Box plot whiskers represent minimum–maximum range. Refer to S7 Fig for comparison between PBS, non-CpG ODN, and CpG-C. The underlying data for this figure can be found in S1 Data, and our gating strategies are provided in S9 Fig. FACS, fluorescence-activated cell sorting; IUDR, iododeoxyuridine; ODN, oligodeoxynucleotide.

Prism (version 7.0c) and Python (version 3.6.3) were used for statistical analysis. When appropriate, the Kolmogorov–Smirnov normality test was used to determine normal distribution of the data, and the F-test or Brown-Forsythe test for determining homogeneity of variance. For normally distributed data with equal variance, we used one-way ANOVA (Fig 5A–5G and 7B, S7 Fig), two-way ANOVA (Figs 1C.i, 1E.i, 3B and 6E and S2, S4, S8 Figs), two-tailed unpaired Student *t* test (Figs 1D, 1E.ii, 1G, 4A–4C, 5H, 5I and 6H, S1 and S2 Figs), or one-tailed unpaired Student *t* test (Fig 8) to compare experimental groups. For normally distributed data with unequal variance, we used Mann–Whitney *U* test (Figs 1C.ii, 1F, 6F and 6G, S1 and S3 Figs) or Kruskal–Wallis (S3 Fig) to compare experimental groups. For non-normally distributed data, we used two-way permutations (Figs 2, 7A and 7C) to compare experimental groups. For post hoc analysis, multiple comparisons were corrected using Dunn test, Tukey, or Bonferroni, according to the primary analysis and the software’s recommendation. For quantification of primary tumor growth dynamics (S2 Fig) and for longitudinal BBB leakage (Fig 4E and 4F), we applied a least squares fit model of an exponential growth curve or one-phase exponential decay curve, respectively, and compared fits of the treated and control groups. The *p*-values smaller than 5% were considered significant. In all experiments, measurements were taken from distinct samples (different animals for in vivo experiments and different wells for in vitro experiments).

## Results

### Prophylactic CpG-C treatment reduces brain metastases in experimental and spontaneous metastases models

To study the prophylactic efficacy of the TLR9 agonist CpG-C in reducing brain metastasis, we first employed two models of non-small-cell lung carcinoma, given the clinical prevalence of brain metastases in this type of cancer [15,45]. To this end, we used the highly metastatic D122 variant of the syngeneic LLC in C57BL/6 mice [32] and the human xenograft PC14-PE6 cells in athymic nude mice [46]. For exclusive injection of tumor cells to the cerebral circulation, we used a novel approach that we have recently developed and validated—the aECAi (Fig 1A and 1B) [32]—which results in improved targeting of tumor cells to the brain and avoids cerebral blood flow perturbations. A single prophylactic systemic injection of CpG-C was given 24 hours before tumor cell injection. Brain tumor growth was monitored thereafter using in vivo bioluminescence imaging. Animals pretreated with CpG-C displayed reduced cerebral tumor growth in both the syngeneic (*p* = 0.0011; Fig 1C) and the xenograft (*p* < 0.0001; Fig 1E) models, exhibiting a statistically significant difference starting on days 14 and 4, respectively. At end point, signal intensity, which is indicative to the tumor burden, was 77-fold lower in the CpG-C–treated mice in the syngeneic D122 model (*n* = 6; *p* < 0.0001) and 82-fold lower in the xenograft model (*n* = 7; *p* < 0.0001), compared with their matching control groups (*n* = 7 in both models). To assure that the differences between groups originated from tumor growth within the brains, rather than from extracranial growth [32], we harvested the brains and measured the tumor signal in both models. In the syngeneic model, brains from CpG-C–treated animals had a 48-fold lower bioluminescent signal compared with control animals (*p* = 0.0040; Fig 1D). Similarly, in the xenograft model the mean area of fluorescence signal, indicative of brain tumor burden, was significantly smaller in CpG-C–treated animals (*p* = 0.0373; Fig 1F).

To test the efficacy of CpG-C treatment in a context that better resembles the clinical setting, we used a murine model of spontaneous brain metastasis that we have recently established [33]. In this model, mCherry-expressing Ret melanoma cells are injected orthotopically, resulting in growth of a primary tumor in the flank. During the perioperative period—three days before and after primary tumor excision—animals were treated with CpG-C (*n* = 15) or vehicle (*n* = 11), with no measurable impact on primary tumor growth (*p* = 0.6066 for tumor growth dynamics and *p* = 0.9260 for tumor size at time of excision; S2 Fig). Approximately nine weeks after excision of the primary tumor, brain and lung metastatic burden (i.e., mCherry expression) were quantified. CpG-C treatment significantly reduced the overall metastatic burden in the brain (*n* = 9 and 12 for control and CpG-C, respectively; *p* = 0.0345; Fig 1G). Notably, in the lungs (as in the primary tumor), CpG-C treatment had no effect (*p* = 0.7858; S2 Fig), suggesting that the beneficial effects of CpG-C in the brain were not secondary to generic or peripheral effects on tumor burden. These results provide direct evidence that systemic prophylactic CpG-C treatment during the perioperative period can reduce metastatic growth in the brain.

### Prophylactic CpG-C is effective in reducing tumor seeding in the brain in a variety of treatment settings

In the subsequent experiments, we aimed to pinpoint mechanisms underlying the beneficial effects of CpG-C. We focused on the first 24 hours of tumor colonization in the brain, for the following reasons: (i) a single administration of CpG-C, which we herein found effective, is known to exert immune activation within hours and for up to 72 hours [11]; (ii) bioluminescence imaging indicated a nonsignificant trend for beneficial effects a day following tumor inoculation (S1 Fig); and (iii) tumor cells successfully proliferate to macrometastases only if they extravasate into the brain parenchyma within the first three days [46]. Therefore, to maximize our ability to focus on the first days following CpG-C administration, we administered syngeneic D122 tumor cells in C57BL/6J mice, employing the aECAi approach [32], known for its high temporal inoculation efficiency. Importantly, as we suspected that bioluminescence was not sensitive enough, we assessed brain tumor seeding by measuring radioactive signals of isotope-labeled tumor cells within an entire excised organ—an approach that allows maximal signal-to-noise sensitivity.

A single prophylactic injection of CpG-C resulted in reduced brain tumor retention similarly in males and females (S3 Fig) and in young, juvenile, and old mice (S3 Fig). While CpG-C was effective in reducing brain tumor retention already at a dose of 1.2 mg/kg (*p* = 0.0455), its efficacy increased at 4 mg/kg (*p* = 0.0003; S3 Fig)—a dose we previously showed as beneficial in reducing peripheral metastases [39]. In the clinical setting, a prophylactic treatment should rely on a chronic schedule, and therefore, we tested whether a regime of five injections of CpG-C given every other day has similar effects as a single injection, and does not result in tolerance to the effects of the agent. Indeed, CpG-C treatment resulted in reduced tumor retention (*p* = 0.0001; S3 Fig) following both the acute (*n* = 6; *p* = 0.0298) and the chronic (*n* = 6; *p* = 0.0013) treatments, compared with control animals (*n* = 6). Notably, single and multiple CpG-C injections were well tolerated, as indicated by a lack of weight loss compared with control animals (*n* = 6; *p* = 0.2593; S3 Fig), in line with previous reports [11]. These data suggest that CpG-C is efficient both as an acute and as a chronic prophylactic treatment for brain metastasis in both sexes and across ages, affecting early stages of tumor cell seeding.

### NK cells and macrophages are not involved in the metastatic process in the brain, nor mediate the beneficial effects of CpG-C

It has previously been shown that CpG-ODNs have beneficial effects in the periphery, reducing seeding of tumor cells and their subsequent growth. These antitumor effects were found to be mediated by NK cells [12,47] and macrophages [10]. To study in vivo whether these leukocytes also take part in the metastatic process in the brain and mediate the effects of CpG-C, we depleted NK cells and monocytes/macrophages using anti-NK1.1 and clodronate liposomes, respectively (Fig 2). In the lungs, NK depletion resulted in a 5-fold increase in tumor retention (*p* = 0.0001) and partially blocked the beneficial effects of CpG-C (*p* = 0.0038; Fig 2A) evident in naïve animals (*p* = 0.0019) (in line with previous results [48]). In contrast, in the brains of the same animals, NK depletion did not affect tumor retention (*p* = 0.3935), nor did it mediate the beneficial effects of CpG-C (*p* = 0.0811; Fig 2B),evident in both naïve (*p* < 0.0001) and NK-depleted animals (*p* = 0.0056). Similarly, depletion of monocytes increased lung (*p* = 0.0401; Fig 2C) but not brain tumor retention (*p* = 0.3081; Fig 2D), while the effects of CpG-C were not mediated by monocytes in the lungs (*p* = 0.0003) or in the brain (*p* = 0.0001). These findings demonstrate that NK cells and monocytes/macrophages play a key role in the metastatic process in the lungs, but not in the brain, nor do they mediate the beneficial effects of CpG-C in the brain.

### CpG-C is taken up by cerebral cells without disrupting BBB integrity

As peripheral innate immune cells do not seem to mediate the effects of CpG-C, we turned to evaluate the role of central nervous system cells that express TLR9 [23,49]. We focused on cells that are known to play key roles in the metastatic process, including endothelial cells, astrocytes, and microglia [50]. First, to evaluate whether CpG-C can cross the BBB and affect cerebral components, we systemically administered mice with TAMRA- or FITC-conjugated CpG-C. Twenty-four hours later, we analyzed CpG-C uptake by brain endothelia, astrocytes, and microglia in histological sections (Fig 3A) and using ImageStream FACS analysis (see methods; Fig 3B). Approximately 74% of endothelial cells, 58% of astrocytes, and 62% of microglia cells internalized CpG-C (*n* = 4 mice; Fig 3B). This internalization is expected, as TLR9 ligands are internalized into the cell to bind with the endosomal receptors [51]. Indeed, lysosomal staining of microglia extracted from CpG-C-TAMRA–treated animals indicated that CpG-C is internalized into the lysosomes (S6 Fig).

For malignant cells to infiltrate into the brain parenchyma, they must cross the BBB. Endothelial cells connected by tight junction act as the first physical barrier, preventing uncontrolled infiltration of blood-borne cells. As endothelial cells uptake CpG-C (Fig 3A and 3B), we sought to test whether it had an effect on BBB permeability and integrity. To this end, we measured biocytin-TMR and IgG infiltration and continuity of claudin-5 (tight junctions) in animals expressing GFP under the claudin-5 promotor. CpG-C did not affect biocytin-TMR or IgG infiltration in the brain vasculature and choroid plexus (≥5 images were averaged in five anatomical regions in three mice—*n* = 12; Fig 4A and 4B S5 Fig), nor continuity of claudin-5 in the brain (Fig 4C, S5 Fig). Furthermore, no infiltration of immune cells (i.e., CD4^+^ or CD68^+^) into the brain or choroid plexus was evident following CpG-C treatment (Fig 4D). Thus, these results strongly suggest that the effects of CpG-C on tumor seeding in the brain are not mediated by perturbations to the BBB or the choroid plexus permeability.

### Microglia, but not astrocytes, mediate antitumor beneficial effects of CpG-C

Astrocytes [52] and microglia [53] have key roles in innate and adaptive immunity, and combined with their significant uptake of CpG-C (Fig 3A and 3B and S6 Fig), they were our primary candidates for mediating the effects of this agent. Therefore, we investigated their in vitro capacity to induce tumor cell lysis and the impact of prestimulation with CpG-C. Primary astrocytic cultures were treated with CpG-C or non-CpG ODN, and tested for their ability to induce tumor cell lysis by contact or by secretion of apoptosis-inducing factors. The cultured astrocytes did not induce tumor cell death, with or without CpG-C treatment, in both contact and secretion conditions (Fig 5A and 5B). In contrast, primary microglial cells induced cytotoxicity in D122 tumor cells, and CpG-C treatment markedly increased this lysis when tumor cells were in contact (Fig 5C), while their conditioned media alone had no effect (Fig 5D). We further extended this testing in the N9 immortalized microglia cell line. Similar to the effects observed in the primary microglia culture, N9 cells reduced tumor cell viability when in contact (Fig 5E), but failed to do so in a paracrine setting (Fig 5F and 5G). To study whether non-CpG ODN impacted the tumoricidal activity of N9 cells, we repeated the contact coculture experiment with an additional group of PBS-treated N9 cultures (S7 Fig). We found PBS and non-CpG ODN treatments to have a similar effect (*p* = 0.7745 and *p* = 0.1420 for 16 × 10^3^ and 32 × 10^3^ D122 cells/well), while CpG-C significantly reduced tumor cells’ viability (for 16 × 10^3^, *p* = 0.0017 and *p* = 0.0062 compared with PBS and non-CpG ODN, respectively, and for 32 × 10^3^, *p* = 0.0003 and *p* = 0.0477 compared with PBS and non-CpG ODN, respectively). Next, we studied the mechanisms by which microglia cells eradicate D122 tumor cells. We found that N9 cells treated with CpG-C induced apoptosis in tumor cells, as indicated by increased annexin V staining (Fig 5H). Additionally, CpG-C treatment resulted in a 3-fold elevation in phagocytosis capacity (Fig 5I), in line with previous reports [27]. Notably, it appears that the effect of CpG-C on microglia activity is not a general activation, as we found no effects of the agent in a scratch migration assay [54] (*n* = 9; *p* = 0.6732 for wound confluency, and *p* = 0.6039 for wound width; and also in vivo as described below, S8 Fig). Taken together, these findings indicate that contact between microglia and tumor cells is essential for the effects induced by CpG-C. A combination of elevated microglial cytotoxicity and enhanced phagocytic capacity underline these effects.

### Microglia mediate the beneficial effects of CpG-C in vivo

**Fig 6:**
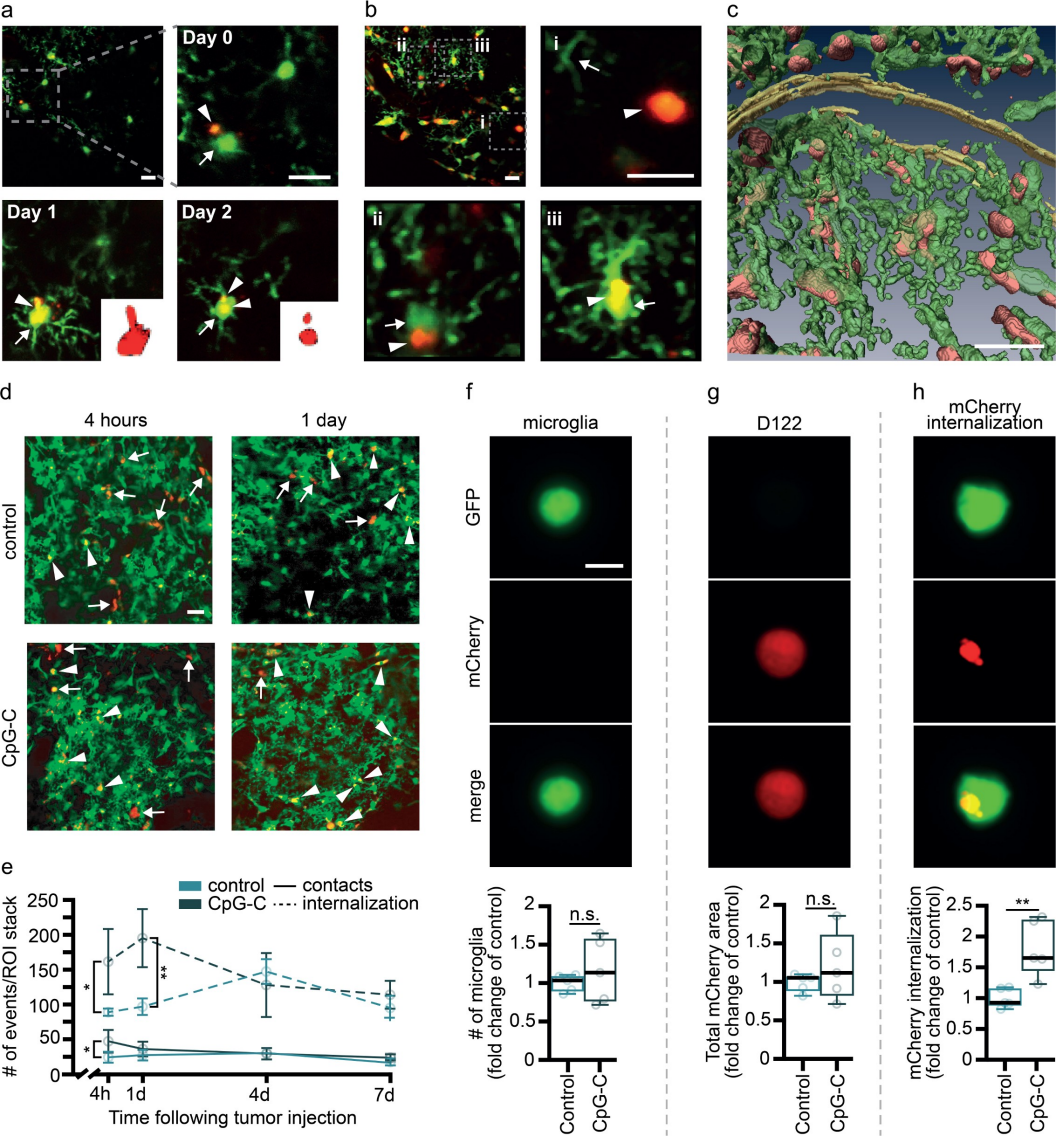
Microglia mediate the in vivo effects of CpG-C. (**a-e**) Chronic in vivo two-photon imaging in CX3CR1^GFP/+^ mice indicated that microglia cells (green) have dynamic relations with tdTomato-labeled D122 tumor cells (red; 15-μm stacks, with 1-μm z-steps) and that CpG-C treatment increases tumor internalization by microglia. (**a**) A microglia cell (arrow) interacting with a tumor cell (arrowhead; two hours post–tumor cells inoculation), phagocytizing it (one day later), and dismantling it (day 2 post–tumor cell injection, inset shows an engulfed tdTomato-positive cell or part of it). (**b**) Different levels of interaction between microglia and tumor cells—(i) no interaction; (ii) contact; and (iii) microglia phagocytized a tumor cell. (**c**) Partial reconstitution of a 15-μm stack with 1-μm z-steps demonstrating the microglia-tumor cells’ “battlefield” four hours after tumor cell injection. (**d**) Representative images and quantification (**e**) of microglia-tumor interactions in control and CpG-C–treated mice four hours and one day following tumor cell inoculation (arrows for contact and arrowheads for internalization). CpG-C treatment resulted in increased contacts four hours following tumor inoculation (*n* = 3; F_(1,4)_ = 2.875, *p* = 0.0218) and in microglia internalization of tumor cells/debris 4 (*p* = 0.0372) and 24 hours (*n* = 3; F_(1,4)_ = 3.400, *p* = 0.0041) following tumor inoculation. Scale bar for (**a-d**) is 20 μm. (**f-h**) CX3CR1^GFP/+^ mice were treated with a single systemic prophylactic CpG-C treatment, injected mCherry-labeled D122 tumor cells using the aECAi approach, and brains were analyzed using ImageStream FACS. While CpG-C treatment did not affect the number of microglia cells (*n* = 5; two-tailed Mann–Whitney *U* = 10, *p* = 0.6905; **f**) or capacity of tumor cell infiltration (indicated by total mCherry area in perfused brains; two-tailed Mann–Whitney *U* = 9, *p* = 0.5476; **g**), it resulted in increased phagocytosis of tumor cells by microglia (two-tailed Student *t* test for unpaired samples, *t*_(4)_ = 3.8850, *p* = 0.0178; **h**). Scale bar for (**e-g**) is 5 μm. Data in (**e**) are presented as mean (±SEM) and box plot whiskers represent minimum–maximum range (**f-h**). The underlying data for this figure can be found in S1 Data. aECAi, assisted external carotid artery inoculation; FACS, fluorescence-activated cell sorting; GFP, green fluorescent protein; n.s., nonsignificant; ROI, region of interest.

**Fig 7:**
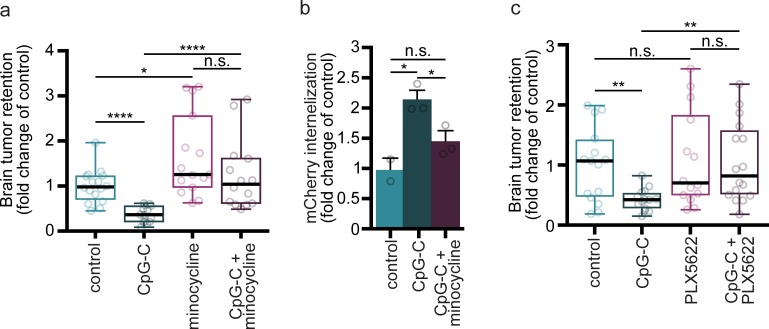
Blocking microglia activation or complete depletion hinders the effects of CpG-C on brain metastasis. (**a-b**) Microglia activation was blocked in vivo using systemic treatment with minocycline. (**a**) Minocycline treatment resulted in increased brain tumor retention of D122 cells (*n* = 15–16, *t*_(27)_ = 66.3229, *p* = 0.0118), while CpG-C treatment reduced tumor retention in naïve mice (*n* = 16 for control and *n* = 14 for minocycline-treated mice; *t*_(28)_ = 63.0149, *p* < 0.0001), but not in minocycline-treated animals (*n* = 14–15; *t*_(27)_ = 42.7850, *p* = 0.1863). The effects of CpG-C were completely blocked by minocycline treatment (*n* = 14; *t*_(26)_ = 86.5528, *p* < 0.0001), indicating that microglia activation mediates the beneficial effects of CpG-C. (**b**) ImageStream FACS analysis indicated that minocycline blocked (*p* = 0.0493) the beneficial effects of prophylactic CpG-C treatment (*p* = 0.01) on the ability of microglia to phagocytize tumor cells (*n* = 2 for control and *n* = 3 for CpG-C and CpG-C + minocycline animals; two-tailed one-way ANOVA with Tukey multiple comparisons test; F_(2,5)_ = 12.85, *p* = 0.0107). (**c**) Without stimulation with CpG-C, microglia cells do not affect brain tumor seeding, as indicated by depletion of microglia cells using the colony stimulating factor 1 receptor inhibitor, PLX5622. Microglia depletion did not affect D122 tumor retention in the brain (*n* = 14–15; *t*_(27)_ = 0.0851, *p* = 0.7490), while it blocked the beneficial effects of CpG-C (*n* = 14 for depleted animals and *n* = 16 for depleted animals treated with CpG-C; *t*_(28)_ = 0.0460, *p* = 0.8637), evident in naïve animals (*n* = 14–15; *t*_(27)_ = 0.0460, *p* = 0.0087). Accordingly, microglia-depleted animals treated with CpG-C had increased brain tumor retention compared with naïve animals treated with CpG-C (*t*_(28)_ = 0.5417, *p* = 0.0068). Two-way permutations were used for analyses of (**a**) and (**c**). Box plot whiskers represent minimum–maximum range (**a,c**), and data in (**b**) are presented as mean (±SEM). The underlying data for this figure can be found in S1 Data. FACS, fluorescence-activated cell sorting; n.s., nonsignificant.

We found that CpG-C affects brain tumor retention as early as 24 hours post–tumor cell inoculation. Interactions between microglia and tumor cells at early stages of tumor cell extravasation have been reported elsewhere [55]. However, the significance of these interactions with respect to microglial tumoricidal characteristics at this time point is yet unknown. To this end, we first established that microglia indeed phagocytize tumor cells at this early time point. Longitudinal intravital imaging revealed that microglia cells interact with tumor cells and initiate phagocytic processes as early as a few hours after tumor cell inoculation (Fig 6A–6C). To assess the effects of CpG-C on this phagocytic capacity, CX3CR1^GFP/+^ mice were injected with CpG-C or non-CpG ODN, and 24 hours later injected with either tdTomato-labeled or mCherry-labeled D122 tumor cells for two-photon or ImageStream FACS analysis, respectively. The numbers of microglia-tumor cell contacts and microglia internalization of mCherry particles (originated from tumor cells) were quantified four hours following tumor cells’ inoculation and at days 1, 4, and 7 thereafter (Fig 6D and 6E). As early as four hours following tumor cells’ inoculation, there were more contacts between microglia and tumor cells in CpG-C–treated animals (*p* = 0.0128), with no differences at later times. Moreover, the number of internalization events in CpG-C–treated animals was higher four hours (*p* = 0.0372) and one day (*p* = 0.0041) following tumor cell inoculation. No differences were evident at days 4 and 7, probably due to the dismantling process of the tumor cells, evident as early as two days following tumor cell inoculation (Fig 6A). Using ImageStream FACS analysis 24 hours after tumor inoculation, we found first that CpG-C did not affect the total number of microglia in the brain (*n* = 5; *p* = 0.4201; Fig 6F) nor the total number of infiltrating tumor cells (*p* = 0.3455; Fig 6G), in accordance with our above findings regarding the lack of CpG-C impact on BBB permeability. However, CpG-C increased phagocytosis of tumor cells by microglia (*p* = 0.0055; Fig 6H). These results alone do not specify whether CpG-C increases the killing of tumor cells by microglia or whether it merely increases endocytosis of tumor debris by microglia. To distinguish between these alternatives, we turned to a set of experiments in which microglia activation was impaired or microglia were depleted from the brain, and quantified the ability of CpG-C to reduce the total amount of live tumor cells by assessing the radioactive signaling that originated from radiolabeled tumor cells. Employing this approach, animals were treated with minocycline, an inhibitor of microglial activation [40,56] (Fig 7A), which resulted in a significantly increased brain tumor retention (*p* = 0.0118), without affecting the total number of infiltrating tumor cells (see below). Importantly, CpG-C treatment reduced tumor retention in naïve mice (*p* < 0.0001), but not in minocycline-treated animals (*p* = 0.1863). Moreover, the effects of CpG-C were completely blocked by minocycline treatment (*p* < 0.0001), indicating the mediating role of microglia in the beneficial effects of CpG-C. To further validate these significant results, animals were treated with CpG-C, or with minocycline and CpG-C, and mCherry (tumor cells) uptake by microglia was quantified using ImageStream FACS analysis and compared with saline-treated animals (Fig 7B). In line with the radioactive-based quantification, CpG-C increased tumor cell phagocytosis (i.e., events in which the mCherry signal could be identified inside GFP-positive segmented objects; *p* = 0.0100), and this effect was blocked by minocycline (*p* = 0.0493). Notably, infiltration capacity of tumor cells was not affected by minocycline treatment, as indicated by total area of mCherry (i.e., all detection events combined) in the brain (*p* = 0.8994). Depletion of all microglia (activated and nonactivated) with PLX5622 [41], a CSF1R inhibitor, blocked the beneficial effects of CpG-C (*p* = 0.0068), again indicating the mediating role of microglia. Microglia depletion alone did not affect tumor retention in brains of naïve animals (*p* = 0.7490; Fig 7C).

**Fig 8:**
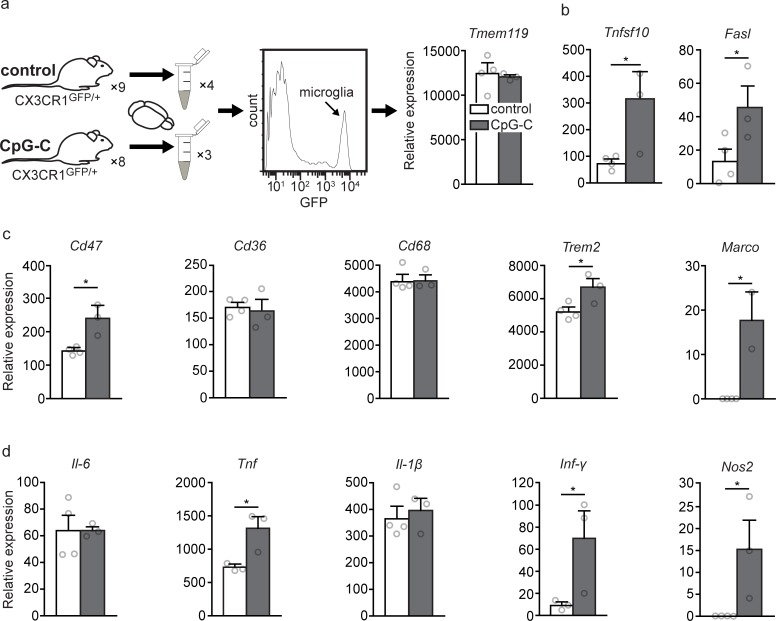
CpG-C treatment results in elevated in vivo expression of apoptosis-inducing, phagocytosis-related, and inflammatory factors. (**a**) CX3CR1^GFP/+^ mice were injected with CpG-C or vehicle, and 24 hours later mRNA expression levels in sorted microglia cells were quantified using RT-qPCR. In one experiment, six animals of each group were pooled into a single sample, and in the second experiment, two CpG-C–treated animals and three controls were analyzed separately (*n* = 3–4 from 8–9 animals). As expected, *Tmem119*, a general microglia marker, was unaffected by the treatment (*t*_(5)_ = 0.371, *p* = 0.7258). (**b**) The death ligands, *Tnfsf10* and *Fasl*, were elevated by 3–4-fold by a single CpG-C injection (*t*_(5)_ = 2.564, *p* = 0.0437; and *t*_(5)_ = 2.36, *p* = 0.0324, respectively). (**c**) Expression levels of receptors related to phagocytosis were significantly higher in microglia of CpG-C–treated animals. While no change was apparent in *Cd36* (*t*_(5)_ = 0.3966, *p* = 0.7080) and *Cd68* (*t*_(5)_ = 0.01655, *p* = 0.9874), a significant increase was evident in *Cd47* (*t*_(5)_ = 2.819, *p* = 0.0186), *Trem2* (*t*_(5)_ = 2.762, *p* = 0.0199), and *Marco* (which was not detected in control animals) (*t*_(4)_ = 4.499, *p* = 0.0108). (**d**) While RNA of the inflammatory cytokines *Il-6* and *Il1-β* was not affected by CpG-C treatment (*t*_(5)_ = 0.04089, *p* = 0.9690; *t*_(5)_ = 0.4417, *p* = 0.6772, respectively), *Tnf* (*t*_(4)_ = 3.207, *p* = 0.0163) and *Inf-γ* (*t*_(4)_ = 2.394, *p* = 0.0374), which synergistically induce apoptosis in tumor cells [54], and *Nos2* (*t*_(5)_ = 2.744, *p* = 0.0203), which is tumoricidal at high concentrations [60], were increased following CpG-C injection. Data are presented as mean (±SEM). The underlying data for this figure can be found in S1 Data, and our gating strategies are provided in S9 Fig. *Cd*, cluster of differentiation; *Fasl*, Fas ligand; GFP, green fluorescent protein; *Il*, interleukin; *Inf-γ*, interferon gamma; *Marco*, macrophage receptor with collagenous structure; *Nos2*, nitric oxide synthase 2; RT-qPCR, real-time quantitative polymerase chain reaction; *Tmem119*, transmembrane protein 119; *Tnf*, tumor necrosis factor; *Tnfsf10*, tumor necrosis factor superfamily member 10; *Trem2*, triggering receptor expressed on myeloid cells 2.

Given our in vitro and in vivo results, we predicted that CpG-C administration would result in elevated expression of apoptosis- and phagocytosis-related factors by microglia cells. We therefore preformed transcriptional analysis of microglia cells isolated from CpG-C–treated or control animals (Fig 8A). We revealed a robust impact of the agent on the induction of mRNA encoding of apoptosis-inducing, phagocytosis-related, and inflammatory factors, while not affecting the inflammation-independent microglial marker *Tmem119* [57] (*p* = 0.7258; Fig 8A). Specifically, mRNA expression of the key apoptosis-inducing ligands, *Tnfsf10* and *Fasl*, increased by 3–4-fold in microglia from CpG-C–treated animals (*p* = 0.0252 and *p* = 0.0324, respectively; Fig 8B). In addition, CpG-C treatment resulted in increased expression of receptors related to phagocytosis [58], including CD47 (*p* = 0.0186) and *Trem2* (*p* = 0.0199), while *Cd36* and *Cd68* mRNA expression levels did not change (*p* = 0.7080 and *p* = 0.9874, respectively; Fig 8C). *Marco*, another important phagocytosis receptor [59], was not detected in microglia of control animals, yet it was highly expressed in CpG-C–treated animals (*p* = 0.0108; Fig 8C). While mRNA of the inflammatory cytokines *Il-6* and *Il1-β* was not affected by CpG-C treatment (*p* = 0.9690 and *p* = 0.6772, respectively), *Tnf* and *Inf-γ*, which are known to synergistically induce apoptosis in tumor cells [54], were increased by approximately 2- and 7-fold, respectively (*p* = 0.0163 and *p* = 0.0374, respectively; Fig 8D). mRNA of *Nos2*, an inflammation-associated enzyme with tumoricidal properties at high concentrations [60], was not detected in control animals, while abundantly expressed in CpG-C–treated animals (*p* = 0.0203; Fig 8D). Irrespectively, and in line with our in vitro results, CpG-C did not affect microglia reaction to a non–tumor-related stimulus in vivo (i.e., laser-induced photodamage; *p* = 0.7474; S8 Fig). Overall, these in vivo findings strengthen the notion that prophylactic treatment with CpG-C is beneficial in reducing brain metastasis by triggering nonactivated microglia cells to adopt tumoricidal characteristics.

## Discussion

**Fig 9:**
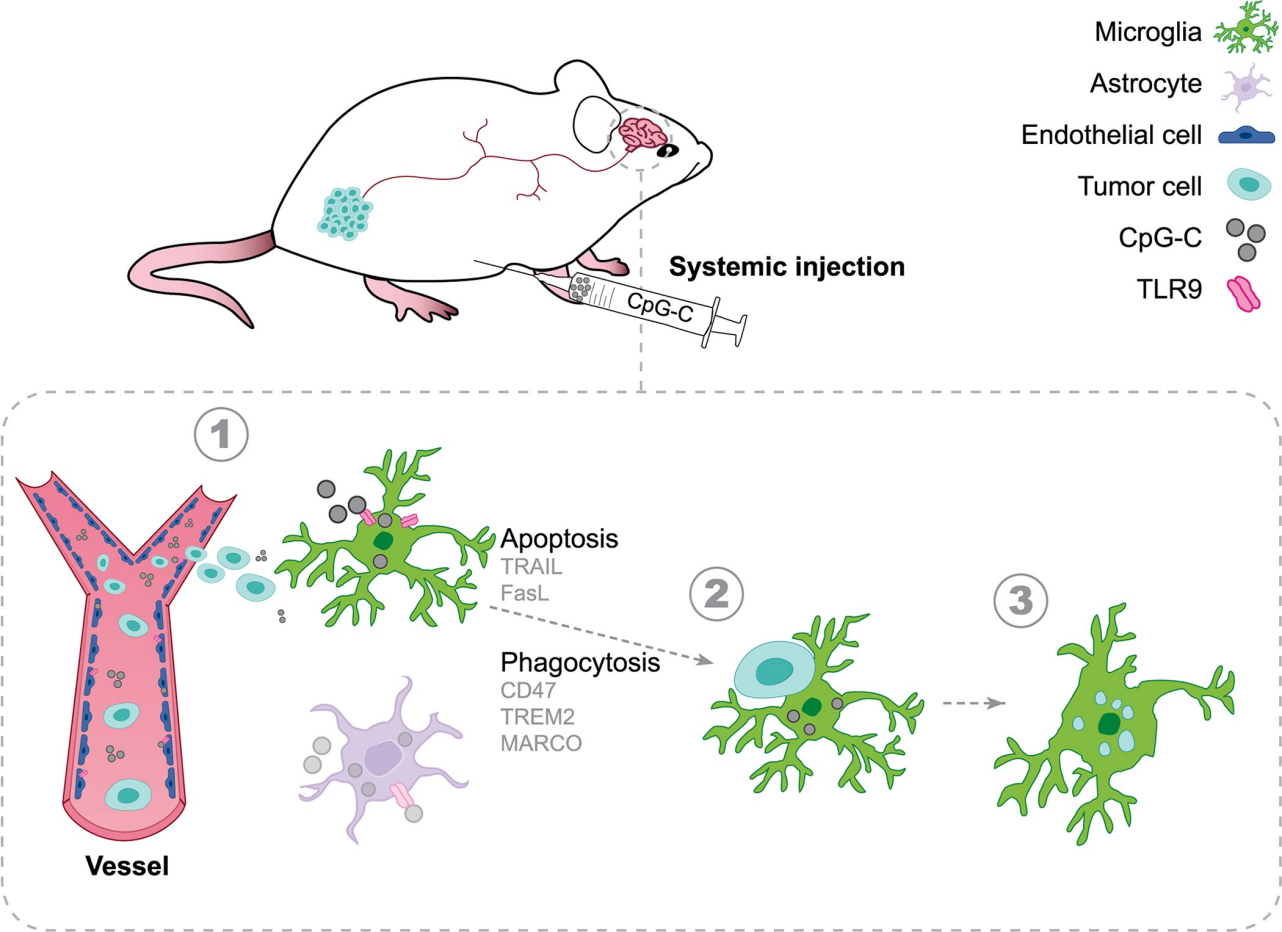
Proposed mechanism. Systemic prophylactic treatment with CpG-C during the perioperative period activates microglia to induce apoptosis in tumor cells and phagocytize them, resulting in reduced brain metastases colonization. A few weeks to months may pass from the time of cancer diagnosis to the time of primary tumor excision [95]. During this period, and a few weeks after surgical excision (known as the perioperative period), there is a high risk for developing brain metastasis with terminal consequences. CpG-C, a TLR9 agonist, given as a systemic prophylactic treatment during this crucial period, infiltrates the brain and activates microglia (1), increasing their expression of *Tnfsf10* and *Fasl*, resulting in contact-dependent induced apoptosis of tumor cells (2). Furthermore, *Cd47*, *Trem2*, and *Marco* expression is increased, triggering enhanced microglial phagocytosis and dismantling of tumor cells (3), thereby reducing brain metastasis colonization. CD, cluster of differentiation; FasL, Fas ligand; Marco, macrophage receptor with collagenous structure; TLR9, toll-like receptor 9; Tnfsf10, tumor necrosis factor superfamily member 10; TREM2, triggering receptor expressed on myeloid cells 2.

Brain metastasis is a detrimental manifestation of cancer progression with limited treatments, and a better understanding of this process is expected to improve therapeutic interventions. Here, employing three tumor models, we report that prophylactic systemic treatment with CpG-C, a TLR9 agonist, exerts beneficial effects through reducing tumor cell seeding and growth in the brain. Notably, NK cells and monocytes did not mediate antimetastatic processes in the brain, nor the beneficial effects of CpG-C, in contrast to their important role in the periphery (shown also here in the lungs). Instead, we identify microglia as key mediators of these beneficial effects in the initial steps of metastatic brain colonization. Moreover, we show that activation of microglia is essential for its antimetastatic function. Thus, CpG-C stimulates microglia to adopt antitumor characteristics, inducing tumor apoptosis and phagocytosis, thereby reducing the formation of brain metastases (Fig 9).

Systemic treatment against brain metastasis has been proposed as a first therapeutic choice [2,4,61], but no effective clinical routine is yet available. A previous study indicated that systemic administration of a CpG-ODN can result in altered cerebral mRNA expression profile [62], suggesting that the agent could have reached this organ. Furthermore, CpG-ODN was shown to stimulate BV2 microglia cells in vitro [63], and intracranial injection of CpG-ODN resulted in activation of microglia cells in vivo [64]. However, there was no direct in vivo evidence demonstrating that such an agent could enter the brain parenchyma if administered systemically and elicit a beneficial effect, fundamental requirements for a prophylactic treatment in cancer patients. Notably, direct intracranial injection of tumor cells or CPG-ODN (or any other agent) alter the neuro-immune environment by eliciting an inflammatory response [65]; thus, interpreting the role of immune cells in these settings is less straightforward. We overcome these technical hurdles and show here, for the first time, that following systemic administration (i.e., intraperitoneally), CpG-C was abundantly taken up by TLR9-expressing cells across the brain without affecting BBB integrity or infiltration of immune cells into the brain (Fig 4, S5 Fig), and dramatically reduced brain colonization by circulating tumor cells (Figs 1,2,6 and 7, S2 and S7 Figs). These findings pave the road for exploiting this compound in the clinic, as it could be easily administered systemically to serve as a prophylactic agent for patients with high risk of developing brain metastases.

An even more urgent clinical scenario in which this treatment could prove life saving is the perioperative period—days to weeks before and after tumor excision—which is now acknowledged as a critical therapeutic window for reducing postoperative metastatic disease [14,66]. Indeed, various short perioperative interventions were reported to markedly impact short- and long-term cancer outcomes [14,67–69]. As brain metastases are common in cancer patients and are associated with poor prognosis [1], reducing their postoperative occurrence is key in improving survival [4]. Here, we show that in a spontaneous brain metastasis model of melanoma, a short perioperative treatment with CpG-C, spanning three days prior to and following primary tumor excision, results in reduced brain tumor burden (Fig 1G). Importantly, CpG-C was shown to have negligible toxicity in humans [19–21]. While we did not directly test whether systemic CpG-C administration has any deleterious effects on neuronal activity, it has been shown by others that when administered directly into the brain (resulting in higher local concentrations), CpG-ODNs do not cause neurotoxicity in animals [27], nor result in significant or permanent neurological deficits in humans [19–21]. Therefore, while traditional chemo- and radiation therapies cannot be used during the perioperative period (due to their deleterious effects on tissue healing and immune competence), the use of CpG-C could be a promising prophylactic approach during this critical time frame [14].

In preclinical trials, acute and chronic systemic CpG-ODNs (including CpG-C) were shown to reduce primary tumor growth and metastases in peripheral organs [10–12,70]. Importantly, CpG-ODNs are evaluated as standalone antitumor agents as well as vaccine adjuvants in several clinical trials of different cancers, and systemic administration is considered well tolerated, with negligible toxicity [71,72]. Given the low toxicity of CpG-C and its wide-range antitumor effects, extended use beyond the perioperative period can also be considered. Additionally, TLR9 stimulation of microglia cells has also been shown to be beneficial in various neurological pathologies, including Alzheimer [26] and seizure-induced aberrant neurogenesis [28], although systemic treatment has not been studied for these conditions. As such, systemic CpG-C treatment could be considered as a therapeutic intervention for cancer and non–cancer-related pathologies.

It is well established that innate immune cells play a key role in preventing and eradicating metastases in the periphery [73–75]. Indeed, we herein show that depletion of NK cells and monocytes results in elevated tumor seeding in the lungs (Fig 2A and 2C). However, in the brains of the same animals, we made the novel observation that NK and monocyte depletions have no effect, and that they do not mediate the beneficial effects of CpG-C (Fig 2B and 2D). While mature NK cells are abundant in the capillaries of the lungs and liver [76], only limited numbers of immature NK cells are found in cerebral capillaries [77]. Also, while patrolling monocytes [21] and pulmonary-resident macrophages are the first line of defense in the lungs [78], monocytes infiltrate the brain parenchyma only under pathological conditions in which the BBB is compromised [79,80], a condition that does not characterize the early stages of tumor cell infiltration [81]. Notably, systemic CpG-C administration did not affect infiltration of T cells (i.e., CD4^+^) or monocytes (i.e., CD68^+^) into the brain (Fig 4D), or the number of GFP^+^ cells (i.e., monocytes/microglia) evident in the brain 24 hours following administration of tumor cells (Fig 6F). These differences between the periphery and the brain underscore the importance of studying brain-specific mechanisms that regulate the metastatic process, to allow tailoring of relevant therapies.

In the brain, microglia are the primary immune effector cells [53]. Close interactions between macrophages/microglia cells and established metastases have been reported in human brain samples [82,83]. In mice, it has been shown that heterogeneous microglia cells, activated and nonactivated, accumulate proximal to invading tumor cells [55] and infiltrate established metastases generated by intracranial injection [84]. However, the role of microglia in regulating brain tumor progression, especially during the initial steps of tumor colonization, remains unclear [83,85–88]. Here, we show that during this time frame (as early as the first 24 hours following administration of tumor cells), CpG-C activates microglia to eradicate micrometastases. Notably, established tumors can modulate activation of microglia, recruiting them to support tumor progression, whereas enabling microglia activation has an opposite effect [87–90]. Therefore, while not directly tested, CpG-C may have also had a secondary effect on development of macrometatases. Our in vitro results indicate that both primary cultured and N9 microglia cells exert low tumoricidal activity (Fig 5), in line with previous findings [91]. Here, however, we clearly show that activation of microglia with CpG-C markedly increases this cytotoxic activity, mediated through direct physical contact with tumor cells and not in a paracrine fashion. While it has been argued that microglia cells promote initial steps of colonization of breast tumor cells in vitro and in acute slices [83], we found through in vivo two-photon imaging that microglia contact and phagocytize tumor cells immediately after their infiltration into the brain (Fig 6A–6C), and they do so more abundantly following systemic administration of CpG-C (Fig 6D and 6E). Accordingly, CpG-C increased mRNA expression of apoptosis-inducing and phagocytosis-related genes in microglia (Fig 8), without affecting microglia density (Fig 6F). Furthermore, by blocking microglia activation with minocycline (Fig 7A and 7B) and by depleting them with CSF1R inhibitor (Fig 7C), we show that microglia mediate the beneficial in vivo antimetastatic effects of CpG-C. While we cannot rule out the possibility that minocycline affected the tumoricidal activity of macrophages [92], given monocyte depletion did not affect tumor burden or mediate the effects of CpG-C (Fig 2D), it is unlikely that the effects of minocycline were mediated by macrophages. Intriguingly, the complete depletion of microglia cells did not affect brain tumor burden in the first 24 hours (Fig 7C). This could be explained by the fact that all the different subsets of microglia are depleted, both those that could have a tumor supportive and tumoricidal role [88].

The metastatic process involves several steps, including arrest in the brain vasculature; infiltration through the BBB, meninges, and blood-cerebrospinal fluid barriers [93]; and colonization of the brain parenchyma [94]. Although CpG-C could have affected all of these steps in different magnitudes, as endothelial cells and astrocytes also uptake the adjuvant (Fig 3), we clearly show that the pool of metastatic cells infiltrating the brain was not altered (Fig 6G), leading to the conclusion that, even if not directly measured, arrest and infiltration were not significantly affected by CpG-C. Support for this argument comes also from our findings that the permeability of key brain-immune interfaces was not altered (Fig 4 and S5 Fig). Nevertheless, this conclusion does not overrule the potential changes in the infiltration rate of circulating metastatic cells through the brain immune interfaces, a topic for future research.

Overall, we demonstrate that shifting the balance from nonactivated to activated microglia, as with the systemic CpG-C treatment presented herein, results in the killing of invading tumor cells and prevents establishment of brain metastases. Such an approach could lay the foundation for a novel clinical perioperative therapy.

## Supporting information

S1 Fig Bioluminescence fails to identify a significant difference after 24 hours. Animals prophylactically treated with CpG-C showed a nonsignificant trend towards reduced bioluminescent signal in the brain compared with control animals in both the syngeneic D122 (two-tailed unpaired Student *t* test, *t*_(11)_ = 1.763, *p* = 0.1056) and xenograft PC14-PE6 tumor models (*t*_(10)_ = 2.155, *p* = 0.0566). The underlying data for this figure can be found in S1 Data. Notice that a more sensitive analysis using radioactive labeling did find a significant and robust difference after 24 hours (Fig 2B).
(TIF)

S2 Fig Perioperative CpG-C treatment did not affect primary melanoma tumor growth or spontaneous lung metastasis. (**a**) Representative images of melanoma Ret-mCherry primary tumor mass (left panels) and sections (right panels) from control and CpG-C–treated animals. No differences in tumor appearance were evident. (**b-c**) CpG-C treatments (arrows) did not affect primary tumor growth dynamics (F_(2,60)_ = 0.5041, *p* = 0.6066; for Y = Y0×exp(k×X) the 95% confidence intervals are: Y0 = 471.8 to 585.3, k = 0.2890 to 0.4971, and Y0 = 509.0 to 571.1, k = 0.3037 to 0.4089 for control and CpG-C, respectively; **b**). Tumors were excised from control and CpG-C–treated animals at the same size (*n* = 9 and *n* = 12 for control and CpG-C, respectively; two-tailed Mann–Whitney *U* = 52.50, *p* = 0.9260; **c**). (**d**) CpG-C treatment during seven perioperative days did not affect micrometastases in the lung (measured by mCherry mRNA expression; *n* = 9 and *n* = 12 for control and CpG-C, respectively; two-tailed unpaired Student *t* test, *t*_(19)_ = 0.2756, *p* = 0.7858). Data in (**b**) are presented as mean (±SEM) and box plot whiskers represent minimum–maximum range (**c-d**). The underlying data for this figure can be found in S1 Data.
(TIF)

S3 Fig CpG-C is effective in reducing brain tumor retention in both sexes, across ages, in a dose-dependent manner, and both as an acute and as a chronic prophylactic treatment. (**a**) A systemic prophylactic injection of CpG-C reduced brain tumor retention of D122 cells in both male (*n* = 5, two-tailed Mann–Whitney *U* = 0, *p* = 0.0079) and female (*n* = 5–6, two-tailed Mann–Whitney *U* = 1, *p* = 0.0087) mice to a similar degree. (**b**) CpG-C reduced brain tumor retention across ages—6 weeks (*n* = 10, two-tailed Mann–Whitney *U* = 7, *p* = 0.0005); 24 weeks (*n* = 10, two-tailed Mann–Whitney *U* = 8, *p* = 0.0007); and 52 weeks (*n* = 10, two-tailed Mann–Whitney *U* = 2, *p* < 0.0001). (**c**) CpG-C reduced brain tumor retention in a dose-dependent manner (*n* = 10–11, Kruskal–Wallis H = 15.98, *p* = 0.0011), reaching significance at 1.2 mg/kg (*p* = 0.0455), and with higher efficacy at 4 mg/kg (*p* = 0.0003). (**d**) An acute systemic injection of CpG-C one day before tumor cell injection (*p* = 0.0298) was as effective as chronic injections (every other day, starting 10 days before tumor inoculation; *p* = 0.0013) in reducing brain tumor retention (*n* = 6, Kruskal–Wallis H = 12.33, *p* = 0.0001). (**e**) No weight loss was evident in animals receiving either acute or chronic systemic CpG-C treatment (*n* = 6, two-tailed two-way ANOVA; F_(2,17)_ = 1.463, *p* = 0.2593). Box plot whiskers represent minimum–maximum range (**a-d**) and data in (**e**) are presented as mean (±SEM). The underlying data for this figure can be found in S1 Data.
(EPS)

S4 Fig NK and monocyte depletion. (**a**) Anti-NK1.1 injection resulted in >90% depletion of NK cells from the blood compared with IgG control. (**b**) Clodronate liposomes resulted in >85% depletion of monocytes from the blood (top panels), without affecting microglia viability (lower panels). IgG, immunoglobulin G; NK, natural killer.
(TIF)

S5 Fig CpG-C does not affect BBB integrity. Mice (*n* = 3) were treated with a single systemic (i.p.) injection of CpG-C (4 mg/kg), and 24 hours later biocytin-TMR and IgG infiltration and claudin-5 continuity were measured in the cortex, cerebellum, midbrain, and hippocampus (five images for each anatomical region; see Methods). (**a**) A tiled sagittal section of a CpG-C–treated mouse. (**b-d**) CpG-C treatment did not affect blood vessels’ leakiness (F_(1,20)_ = 0.0828, *p* = 0.7765 and F_(1,20)_ = 1.738, *p* = 0.2023 for biocytin-TMR and IgG, respectively; **b-c**) nor claudin-5 continuity (F_(1,11)_ = 0.1272, *p* = 0.7281; **d**) in any of the analyzed brain regions. Scale bar is 50 μm. Data are presented as mean (±SEM). The underlying data for this figure can be found in S1 Data. BBB, blood-brain barrier; IgG, immunoglobulin G; i.p., intraperitoneal; TMR, tetramethylrhodamine.
(TIF)

S6 Fig CpG-C is taken up into microglia lysosomes in vitro and in vivo. (**a**) TAMRA-labeled CpG-C injected systemically is taken up by microglia in vivo in CX3CR1^GFP/+^ mice (top left—before CpG-C injection; bottom left—after CpG-C injection; right panel—partial reconstruction; 15-μm stacks, with 1-μm z-steps). (**b**) N9 cells pretreated with TAMRA-labeled CpG-C for 24 hours (top panels) and microglia cells extracted from CX3CR1^GFP/+^ mice that were injected with TAMRA-labeled CpG-C 24 hours earlier (bottom panels) were costained with Lysotracker, demonstrating CpG-C was taken up into the lysosomes. TAMRA, tetramethylrhodamine.
(TIF)

S7 Fig PBS and non-CpG ODN affect tumor cells’ viability similarly. (**a**) No differences in brain tumor retention were evident between PBS- and non-CpG ODN–treated animals (*p* = 0.9974), while CpG-C significantly reduced brain tumor retention of D122 cells (F_(2,28)_ = 8.277, *p* = 0.0040 and *p* = 0.0048 compared with PBS and non-CpG ODN, respectively). (**b**) D122 cells were cocultured in contact with N9 cells treated with PBS, non-CpG ODN, or CpG-C. No differences in tumor cells’ viability were evident between PBS- and non-CpG ODN–treated cultures (*p* = 0.7745 and *p* = 0.1420 for 16 × 10^3^ and 32 × 10^3^ D122 cells/well), while CpG-C significantly reduced tumor cells’ viability (for 16 × 10^3^: F_(2,20)_ = 9.767, *p* = 0.0017 and *p* = 0.0062 compared with PBS and non-CpG ODN, respectively, and for 32 × 10^3^: F_(2,19)_ = 12.15, *p* = 0.0003 and *p* = 0.0477 compared with PBS and non-CpG ODN, respectively). Box plot whiskers represent minimum–maximum range. The underlying data for this figure can be found in S1 Data. ODN, oligodeoxynucleotide.
(EPS)

S8 Fig CpG-C does not affect microglia reaction to non-tumor–related insults. (**a**) Microglial N9 cultures treated with 100 nM/L CpG-C for 24 hours reacted similarly in the scratch migration assay compared with cultures treated with non-CpG ODN, indicated by wound confluence (F_(1,16)_ = 0.1845, *p* = 0.6732) and wound width (F_(1,16)_ = 0.2801, *p* = 0.6039). Scale bar is 300 μm. (**b**) Microglia reacted similarly to a photodamage induced in vivo by a high-power laser (780 nm; 150 mW at the sample; about 1 μm in size) in CpG-C–treated and control CX3CR1^GFP/+^ mice (F_(1,8)_ = 0.1111, *p* = 0.7474). Scale bar is 50 μm. Data are presented as mean (±SEM). The underlying data for this figure can be found in S1 Data. ODN, oligodeoxynucleotide.
(TIF)

S9 Fig FACS analyses gating strategies. (**a**) Annexin V in vitro experiments (Fig 4H) were analyzed by selecting single cells from a plot of SSC against FSC. (**b-d**) The gates for mCherry-positive cells (D122; **b**) and annexin V–positive cells (**c**) were selected based on samples negative for these stains and validated using positive control samples containing N9 and mCherry-labeled D122 cells (**d**). To induce annexin V staining (indicating apoptosis) in positive control samples, half of the cells were placed in 90°C for 2 minutes and then immediately on ice for 2 minutes, and mixed together. (**e**) Examples for non-CpG ODN–(control; left panel) and CpG-C–treated wells (right panel). (**f**) ImageStream image data files were analyzed by selecting single cells from a plot of object area against object aspect ratio (width/length; left panel) and then focused cells using the Gradient RMS feature (right panel). (**g**) For CpG-C uptake experiments (Fig 3C), cells that have taken up FITC-labeled CpG-C were identified on a scatterplot of FITC against the appropriate fluorophore (e.g., APC for microglia cells). (**h**) For experiments in Fig 5E–5G and 5I, mCherry-positive cells (left panel) and microglia cells (from CX3CR1^GFP/+^ mice; middle panel) were identified on a scatterplot of intensity of the relevant fluorophore against object aspect ratio. mCherry-positive microglia cells were identified inside the microglia subpopulation in a histogram of the of mCherry intensity (right panel). For quantification of (**g**) and (**h**; right panel), we used the internalization wizard. APC, allophycocyanin; FACS, fluorescence-activated cell sorting; FITC, fluorescein isothiocyanate; FSC, forward scatter; ODN, oligodeoxynucleotide; RMS, Ret-mCherry–sorted; SSC, side scatter.
(EPS)

S1 Movie Microglia (white) treated with CpG-C phagocytize invading tumor cells (red) in vivo as early as a few hours after tumor cell inoculation. Orange arrows mark phagocytosis events at day 0 (left) and their corresponding events at day 1 (right). The field of view for each day is 200 μm.
(MP4)

S1 Data Aggregated data used to generate figure panels. (XLSX)

S1 Table Table of antibodies. (XLSX)

## Abbreviations

aECAi: assisted external carotid artery inoculation
APC: allophycocyanin
BBB: blood-brain barrier
CD: cluster of differentiation
CSF1R: colony stimulating factor-1 receptor
EAE: experimental autoimmune encephalomyelitis
ECA: external carotid artery
FasL: Fas ligand
GFP: green fluorescent protein
Il: interleukin
Inf-γ: interferon gamma
i.p.: intraperitoneal
IUDR: iododeoxyuridine
LLC: Lewis lung carcinoma
mAb: monoclonal antibody
Marco: Macrophage receptor with collagenous structure
NK: natural killer
Nos2: nitric oxide synthase 2
ODN: oligodeoxynucleotide
PFA: paraformaldehyde
PoRTS: polished and reinforced thin-skull
RMS: Ret-mCherry–sorted
ROI: region of interest
SCLC: small-cell lung cancer
TAMRA/TMR: tetramethylrhodamine
TLR: toll-like receptor
Tmem119: transmembrane protein 119
Tnf: tumor necrosis factor
Tnfsf10: tumor necrosis factor superfamily member 10
TREM2: triggering receptor expressed on myeloid cells 2
