# Supplementary figures and images for: Prophylactic TLR9 stimulation reduces brain metastasis through microglia activation
Source: PLoS Biol. 2019 Mar 28;17(3):e2006859. doi: 10.1371/journal.pbio.2006859 (PMC6469801; doi:10.1371/journal.pbio.2006859)

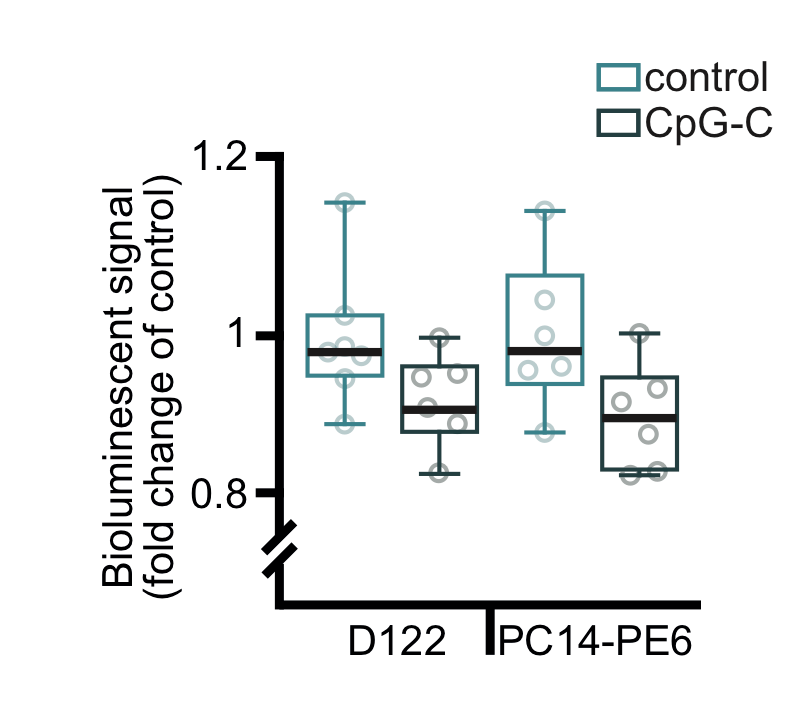

Supplement: S1 Fig — Animals prophylactically treated with CpG-C showed a nonsignificant trend towards reduced bioluminescent signal in the brain compared with control animals in both the syngeneic D122 (two-tailed unpaired Student t test, t(11) = 1.763, p = 0.1056) and xenograft PC14-PE6 tumor models (t(10) = 2.155, p = 0.0566). The underlying data for this figure can be found in S1 Data. Notice that a more sensitive analysis using radioactive labeling did find a significant and robust difference after 24 hours (Fig 2B). (TIF) [file pbio.2006859.s001.tif]

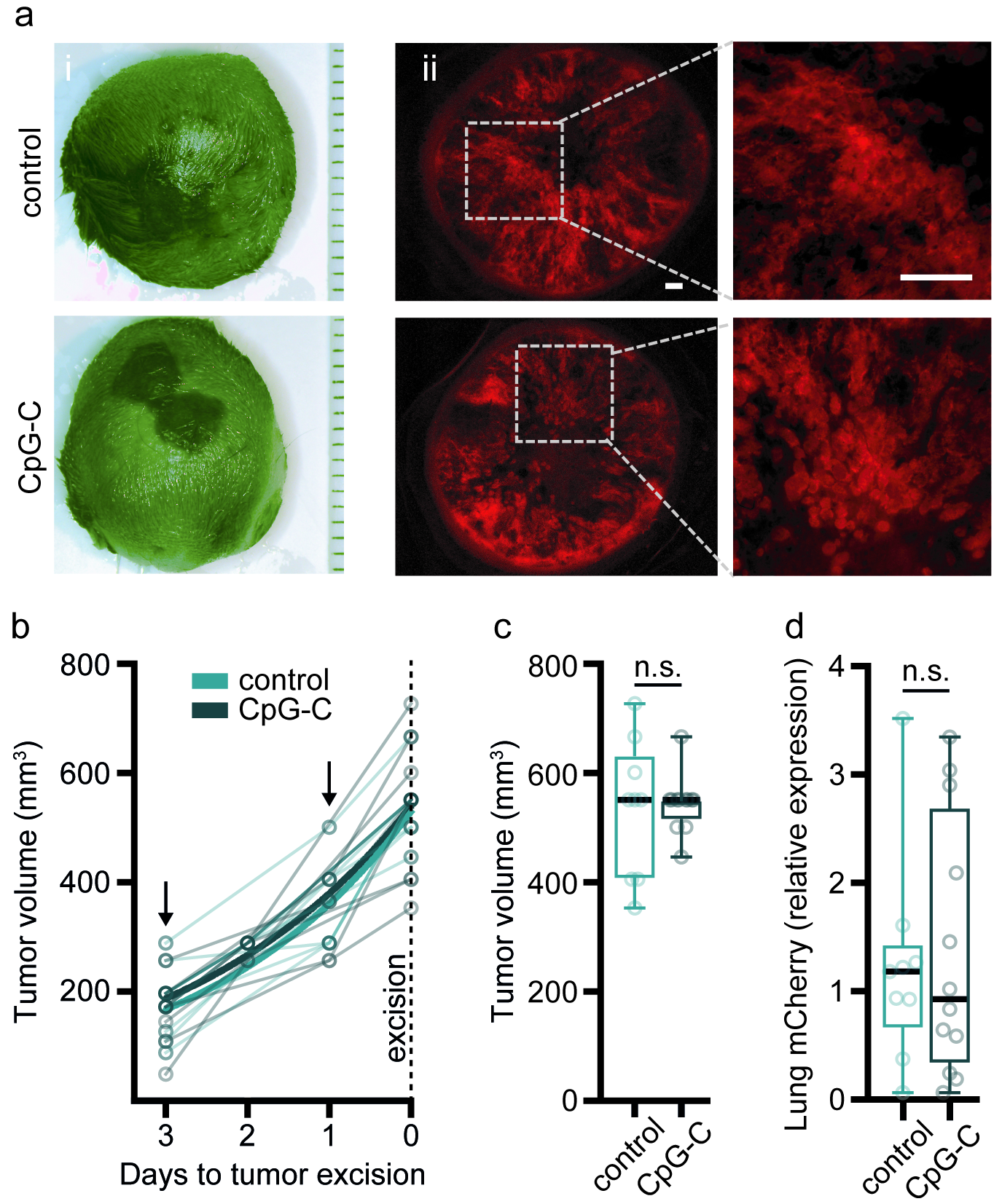

Supplement: S2 Fig — (a) Representative images of melanoma Ret-mCherry primary tumor mass (left panels) and sections (right panels) from control and CpG-C–treated animals. No differences in tumor appearance were evident. (b-c) CpG-C treatments (arrows) did not affect primary tumor growth dynamics (F(2,60) = 0.5041, p = 0.6066; for Y = Y0×exp(k×X) the 95% confidence intervals are: Y0 = 471.8 to 585.3, k = 0.2890 to 0.4971, and Y0 = 509.0 to 571.1, k = 0.3037 to 0.4089 for control and CpG-C, respectively; b). Tumors were excised from control and CpG-C–treated animals at the same size (n = 9 and n = 12 for control and CpG-C, respectively; two-tailed Mann–Whitney U = 52.50, p = 0.9260; c). (d) CpG-C treatment during seven perioperative days did not affect micrometastases in the lung (measured by mCherry mRNA expression; n = 9 and n = 12 for control and CpG-C, respectively; two-tailed unpaired Student t test, t(19) = 0.2756, p = 0.7858). Data in (b) are presented as mean (±SEM) and box plot whiskers represent minimum–maximum range (c-d). The underlying data for this figure can be found in S1 Data. (TIF) [file pbio.2006859.s002.tif]

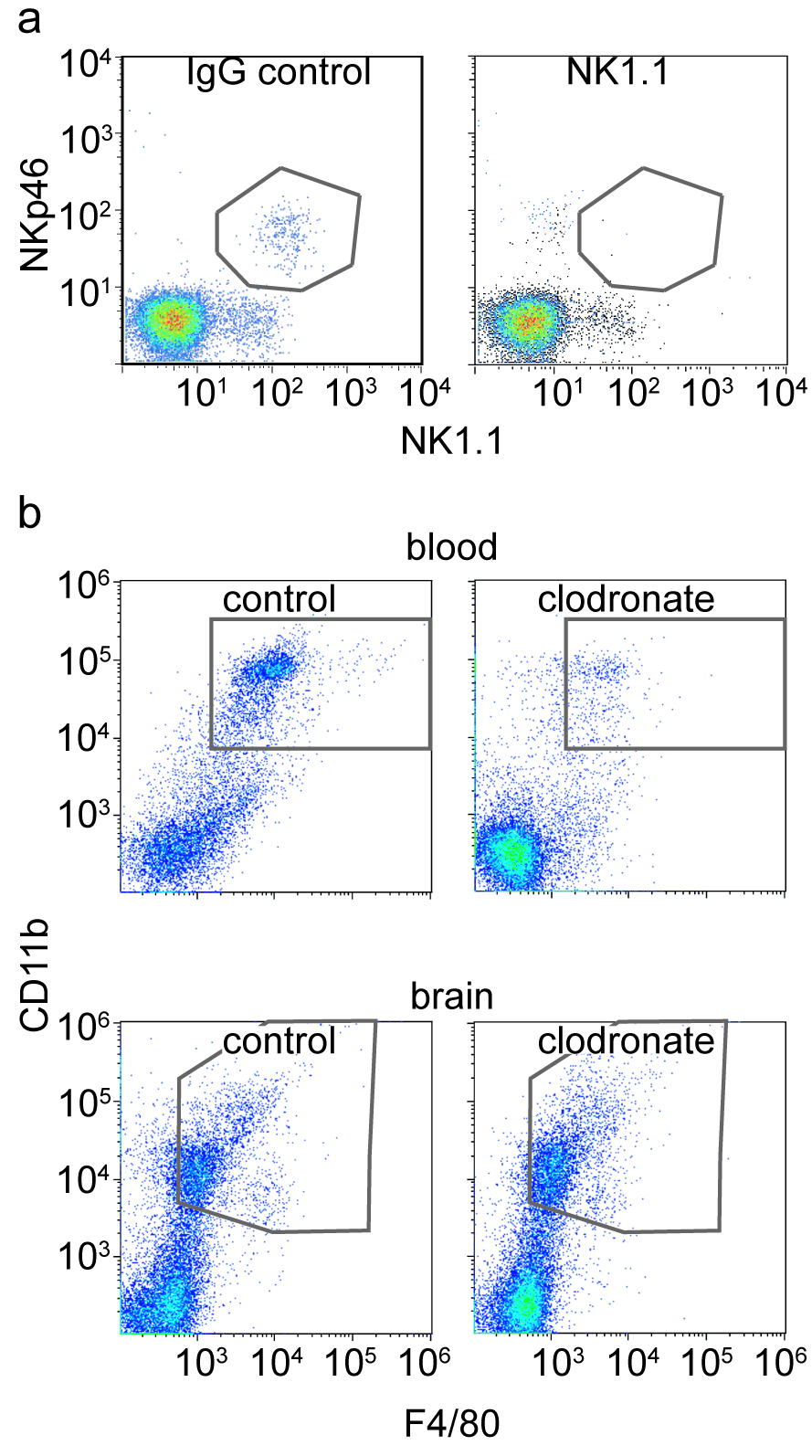

Supplement: S4 Fig — (a) Anti-NK1.1 injection resulted in >90% depletion of NK cells from the blood compared with IgG control. (b) Clodronate liposomes resulted in >85% depletion of monocytes from the blood (top panels), without affecting microglia viability (lower panels). IgG, immunoglobulin G; NK, natural killer. (TIF) [file pbio.2006859.s004.tif]

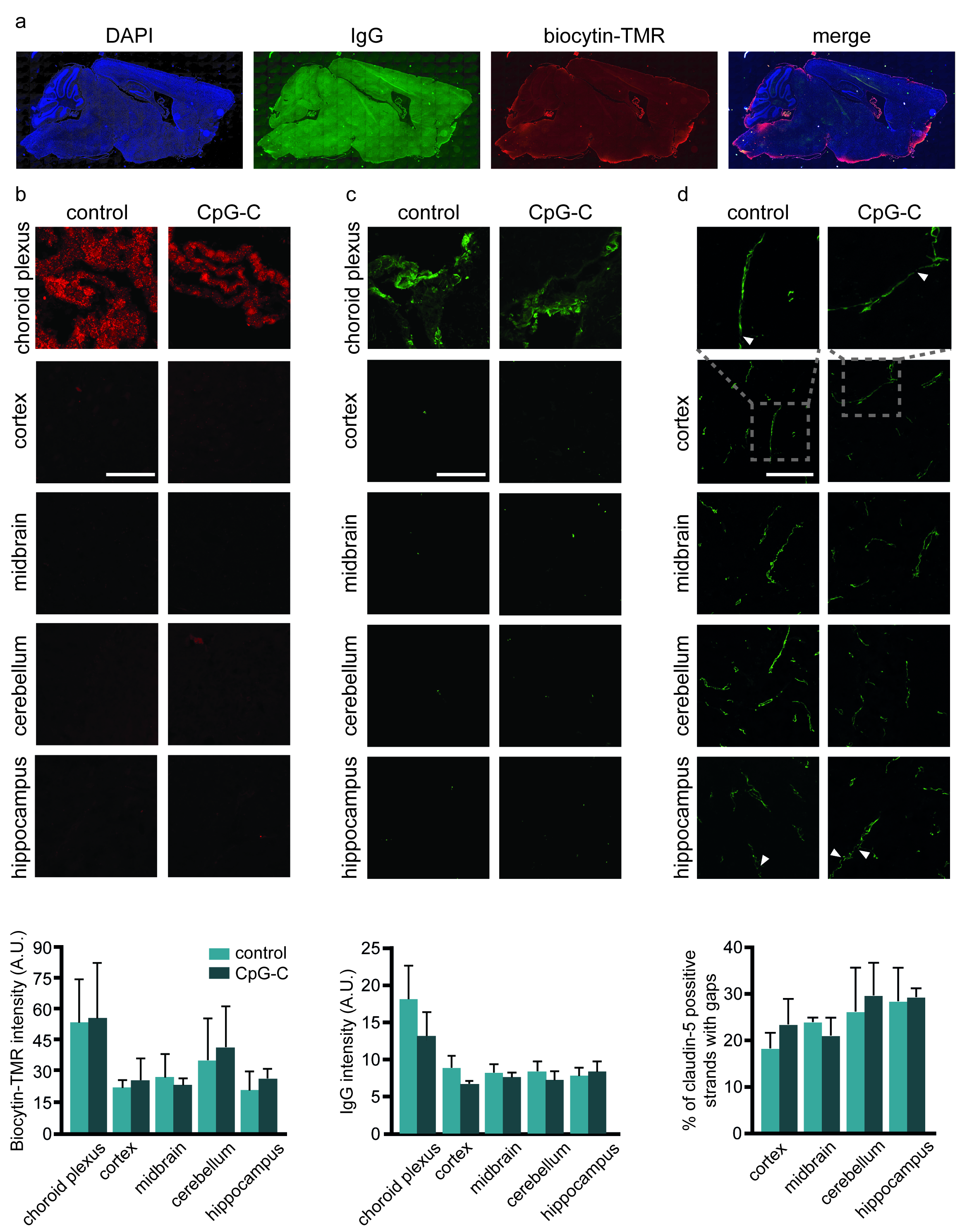

Supplement: S5 Fig — Mice (n = 3) were treated with a single systemic (i.p.) injection of CpG-C (4 mg/kg), and 24 hours later biocytin-TMR and IgG infiltration and claudin-5 continuity were measured in the cortex, cerebellum, midbrain, and hippocampus (five images for each anatomical region; see Methods). (a) A tiled sagittal section of a CpG-C–treated mouse. (b-d) CpG-C treatment did not affect blood vessels’ leakiness (F(1,20) = 0.0828, p = 0.7765 and F(1,20) = 1.738, p = 0.2023 for biocytin-TMR and IgG, respectively; b-c) nor claudin-5 continuity (F(1,11) = 0.1272, p = 0.7281; d) in any of the analyzed brain regions. Scale bar is 50 μm. Data are presented as mean (±SEM). The underlying data for this figure can be found in S1 Data. BBB, blood-brain barrier; IgG, immunoglobulin G; i.p., intraperitoneal; TMR, tetramethylrhodamine. (TIF) [file pbio.2006859.s005.tif]

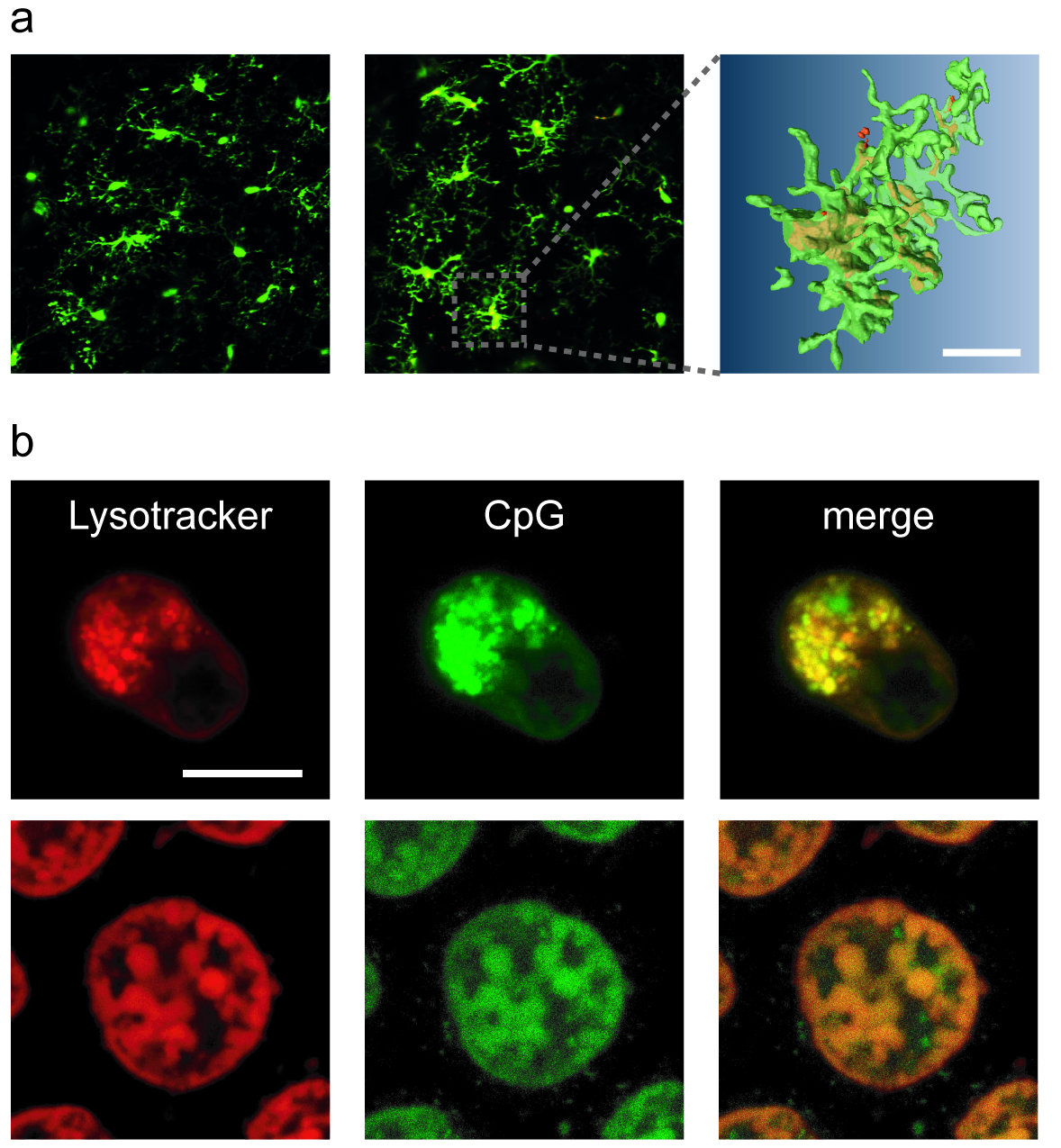

Supplement: S6 Fig — (a) TAMRA-labeled CpG-C injected systemically is taken up by microglia in vivo in CX3CR1GFP/+ mice (top left—before CpG-C injection; bottom left—after CpG-C injection; right panel—partial reconstruction; 15-μm stacks, with 1-μm z-steps). (b) N9 cells pretreated with TAMRA-labeled CpG-C for 24 hours (top panels) and microglia cells extracted from CX3CR1GFP/+ mice that were injected with TAMRA-labeled CpG-C 24 hours earlier (bottom panels) were costained with Lysotracker, demonstrating CpG-C was taken up into the lysosomes. TAMRA, tetramethylrhodamine. (TIF) [file pbio.2006859.s006.tif]

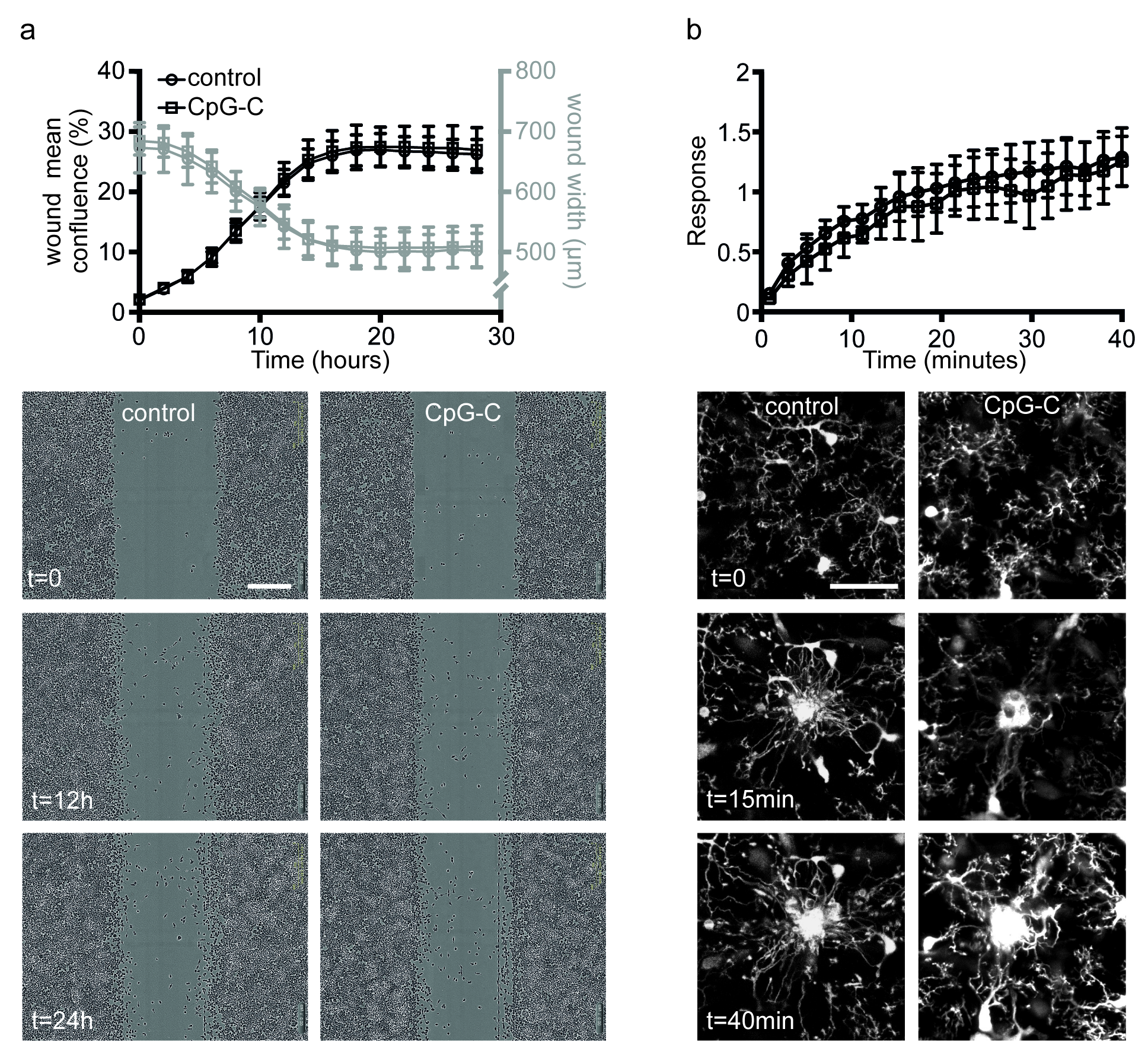

Supplement: S8 Fig — (a) Microglial N9 cultures treated with 100 nM/L CpG-C for 24 hours reacted similarly in the scratch migration assay compared with cultures treated with non-CpG ODN, indicated by wound confluence (F(1,16) = 0.1845, p = 0.6732) and wound width (F(1,16) = 0.2801, p = 0.6039). Scale bar is 300 μm. (b) Microglia reacted similarly to a photodamage induced in vivo by a high-power laser (780 nm; 150 mW at the sample; about 1 μm in size) in CpG-C–treated and control CX3CR1GFP/+ mice (F(1,8) = 0.1111, p = 0.7474). Scale bar is 50 μm. Data are presented as mean (±SEM). The underlying data for this figure can be found in S1 Data. ODN, oligodeoxynucleotide. (TIF) [file pbio.2006859.s008.tif]
